# Supplementary material for: Novel Non-integrating DNA Nano-S/MAR Vectors Restore Gene Function in Isogenic Patient-Derived Pancreatic Tumor Models
Source: Mol Ther Methods Clin Dev. 2020 Apr 25;17:957–68. doi: 10.1016/j.omtm.2020.04.017 (PMC7218229; doi:10.1016/j.omtm.2020.04.017)
Supplement: Document S1. Figures S1–S6 [file mmc1.pdf]

OMTM, Volume *17*

Supplemental Information

Novel Non-integrating DNA Nano-S/MAR Vectors Restore Gene Function in Isogenic Patient-Derived Pancreatic Tumor Models

Matthias Bozza, Edward W. Green, Elisa Espinet, Alice De Roia, Corinna Klein, Vanessa Vogel, Rienk Offringa, James A. Williams, Martin Sprick, and Richard P. Harbottle

Supplementary Figure 1

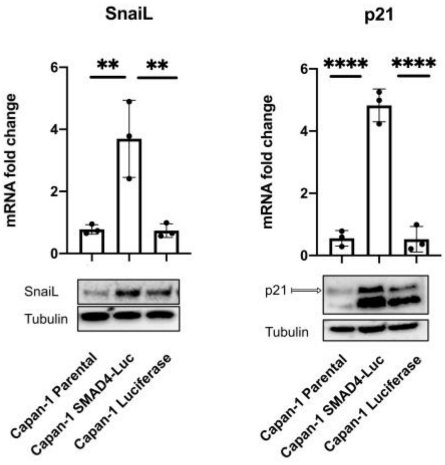

Supplementary Figure 1. Quantitative Real Time PCR analysis of SnaiL and p21 in Capan-1. The expression of SnaiL and p21 was addressed in genetically modified Capan-1 with SMAD4 and Luciferase, in comparison to parental unmodified cells through qRT-PCR and Western Blot.

Supplementary Figure 2

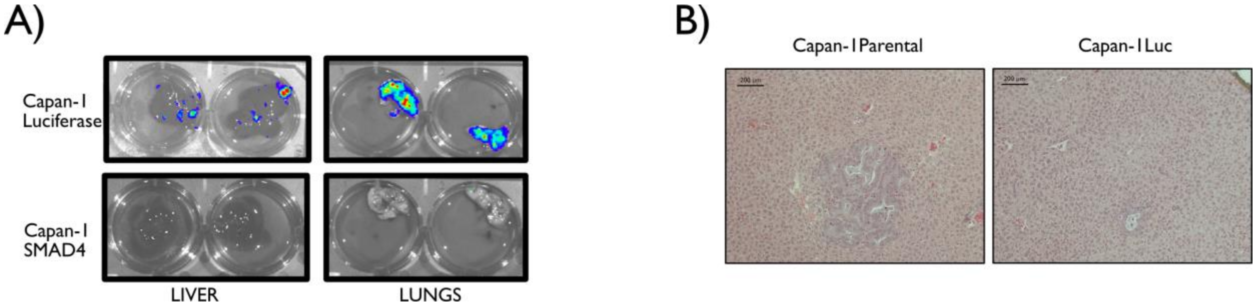

Supplementary Figure 2. Bioluminescent detection of metastasis. (A) The formation of metastasis in the liver and in the lungs of mice injected with Capan-1 labelled with the reporter gene Luciferase were detected through Bio Luminescent Imaging (BLI) imaging and (B) their morphology was assessed through H&E staining.

Supplementary Figure 3

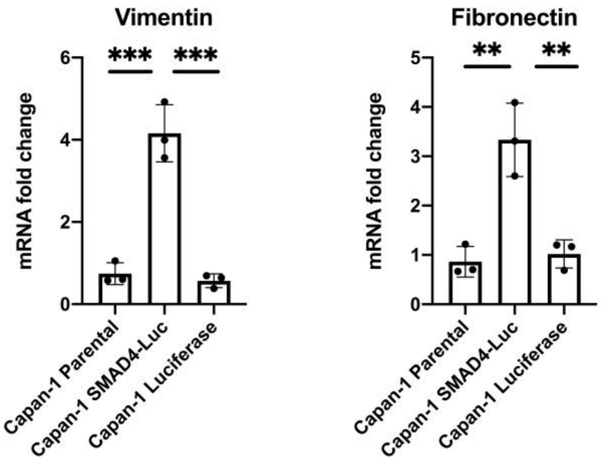

Supplementary Figure 3. Analysis of EMT markers. The expression of Vimentin and Fibronectin in modified and unmodified Capan-1 cells was investigated through qRT-PCR.

Supplementary Figure 4

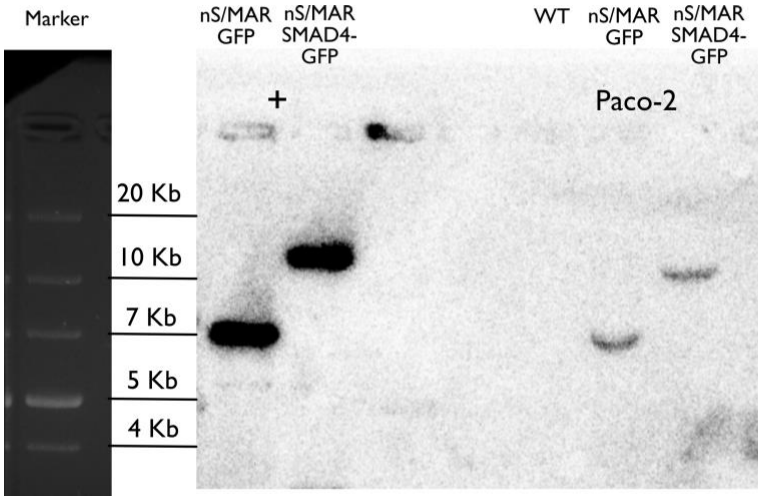

Supplementary Figure 4. Southern Blot analysis of PACO2 cells modified with Nano-S/MAR vectors.

The molecular integrity and the episomal maintenance of the two Nano-S/MAR constructs were evaluated by Southern Blot analysis. Control Nano-Vectors (first two lanes) and the total DNA

from the modified PACO2 cells was digested with the restriction enzyme BamHI and separated on a 1 % agarose gel before being transferred on a nylon membrane. The GFP reporter gene was used to generate the radioactive probe. The plasmid isolated via total DNA extraction from established cells showed an identical size to their respective reference controls. The DNA molecular weight marker is shown as a reference.

Supplementary Figure 5

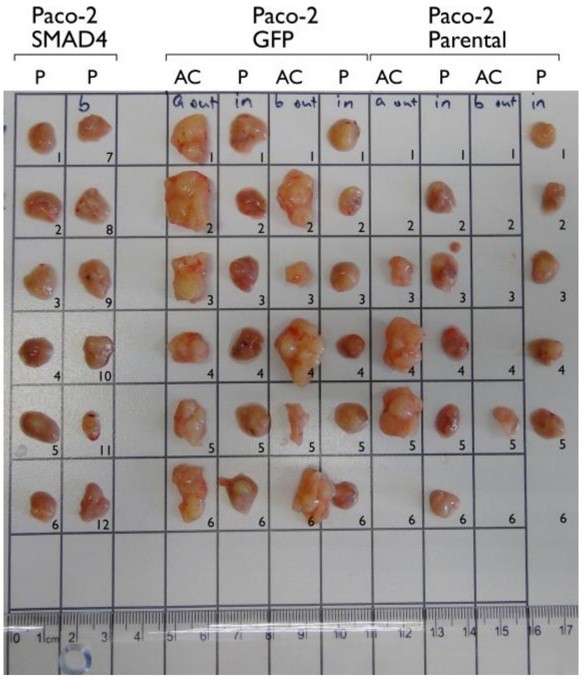

Supplementary Figure 5. PACO2 cells macroscopic evaluation of tumour growth.

PACO2 Parental and GFP cells form tumours in the pancreas (P) and in the abdominal cavity (AC) of the injected mice. Paco-2 SMAD4 cells did not form clear tumour masses in the injection site and abdominal cavity.

Supplementary Figure 6

A) SMAD4 open frame cDNA

ATGGACAATATGTCTATTACGAATACACCAACAAGTAATGATGCCTGTCTGAGCATTGTGCATAGTTTGA
TGTGCCATAGACAAGGTGGAGAGAGTGAAACATTTGCAAAAAGAGCAATTGAAAGTTTGGTAAAGAAG
CTGAAGGAGAAAAAAGATGAATTGGATTCTTTAATAACAGCTATAACTACAAATGGAGCTCATCCTAGT
AAATGTGTTACCATACAGAGAACATTGGATGGGAGGCTTCAGGTGGCTGGTCGGAAAGGATTTCCTCAT
GTGATCTATGCCCGTCTCTGGAGGTGGCCTGATCTTCACAAAAATGAACTAAAACATGTTAAATATTGTC
AGTATGCGTTTGACTTAAAATGTGATAGTGTCTGTGTGAATCCATATCACTACGAACGAGTTGTATCACC
TGGAATTGATCTCTCAGGATTAACACTGCAGAGTAATGCTCCATCAAGTATGATGGTGAAGGATGAATA
TGTGCATGACTTTGAGGGACAGCCATCGTTGTCCACTGAAGGACATTCAATTCAAACCATCCAGCATCCA
CCAAGTAATCGTGCATCGACAGAGACATACAGCACCCCAGCTCTGTTAGCCCCATCTGAGTCTAATGCTA
CCAGCACTGCCAACTTTCCCAACATTCCTGTGGCTTCCACAAGTCAGCCTGCCAGTATACTGGGGGGCAG
CCATAGTGAAGGACTGTTGCAGATAGCATCAGGGCCTCAGCCAGGACAGCAGCAGAATGGATTTACTG
GTCAGCCAGCTACTTACCATCATAACAGCACTACCACCTGGACTGGAAGTAGGACTGCACCATACACACC
TAATTTGCCTCACCACCAAAACGGCCATCTTCAGCACCACCCGCCTATGCCGCCCCATCCCGGACATTACT
GGCCTGTTCACAATGAGCTTGCATTCCAGCCTCCCATTTCCAATCATCCTGCTCCTGAGTATTGGTGTTCC
ATTGCTTACTTTGAAATGGATGTTCAGGTAGGAGAGACATTTAAGGTTCCTTCAAGCTGCCCTATTGTTA
CTGTTGATGGATACGTGGACCCTTCTGGAGGAGATCGCTTTTGTTTGGGTCAACTCTCCAATGTCCACAG
GACAGAAGCCATTGAGAGAGCAAGGTTGCACATAGGCAAAGGTGTGCAGTTGGAATGTAAAGGTGAA
GGTGATGTTTGGGTCAGGTGCCTTAGTGACCACGCGGTCTTTGTACAGAGTTACTACTTAGACAGAGAA
GCTGGGCGTGCACCTGGAGATGCTGTTCATAAGATCTACCCAAGTGCATATATAAAGGTCTTTGATTTGC
GTCAGTGTCATCGACAGATGCAGCAGCAGGCGGCTACTGCACAAGCTGCAGCAGCTGCCCAGGCAGCA
GCCGTGGCAGGAAACATCCCTGGCCCAGGATCAGTAGGTGGAATAGCTCCAGCTATCAGTCTGTCAGCT
GCTGCTGGAATTGGTGTTGATGACCTTCGTCGCTTATGCATACTCAGGATGAGTTTTGTGAAAGGCTGG
GGACCGGATTACCCAAGACAGAGCATCAAAGAAACACCTTGCTGGATTGAAATTCACTTACACCGGGCC
CTCCAGCTCCTAGACGAAGTACTTCATACCATGCCGATTGCAGACCCACAACCTTTAGAC

B) Illumina probe for SMAD4 transcript

GCAGCGTCACTCTACCTAATGTCTCACTGTTCTGCAAAGGTGGCAATGCT

Supplementary Figure 6. Sequences for microarray analysis

A) SMAD4 open reading frame was codon-optimized and stop codon was removed to allow the tandem expression of the tumour suppressor with a reporter gene. This sequence is not recognized by the Illumina probe (B) responsible for the detection of SMAD4 endogenous transcript.

TABLE

Table 1. Differentially expressed genes in Capan-1 Luciferase *vs* Capan-1 unmodified parental cells with a fold change greater than 2 (*n* = 351)

Table 2. Differentially expressed genes in Capan-1 SMAD4 Luciferase *vs* Capan-1 Luciferase cells (*n* = 189 genes)

Table 3. Differentially expressed genes in PACO2 GFP Vs PACO2 Parental cells with a fold change greater than 2 (n = 2)

Table 4. Differentially expressed genes in PACO2 GFP-SMAD4 *vs* PACO2 GFP cells with a fold change greater than 2 (n = 169)

REFERENCES

1. Dinca, EB, Sarkaria, JN, Schroeder, MA, Carlson, BL, Voicu, R, Gupta, N, *et al.* (2007). Bioluminescence monitoring of intracranial glioblastoma xenograft: response to primary and salvage temozolomide therapy. *J Neurosurg* **107**: 610-616.
2. Argyros, O, Wong, SP, Niceta, M, Waddington, SN, Howe, SJ, Coutelle, C, *et al.* (2008). Persistent episomal transgene expression in liver following delivery of a scaffold/matrix attachment region containing non-viral vector. *Gene Ther* **15**: 1593-1605.
3. Argyros, O, Wong, SP, Gowers, K, and Harbottle, RP (2012). Genetic modification of cancer cells using non-viral, episomal S/MAR vectors for in vivo tumour modelling. *PLoS One* **7**: e47920.
4. Wong, SP, Argyros, O, Coutelle, C, and Harbottle, RP (2011). Non-viral S/MAR vectors replicate episomally in vivo when provided with a selective advantage. *Gene Ther* **18**: 82-87.
5. Argyros, O, Wong, SP, and Harbottle, RP (2011). Non-viral episomal modification of cells using S/MAR elements. *Expert Opin Biol Ther* **11**: 1177-1191.
6. Stehle, IM, Postberg, J, Rupprecht, S, Cremer, T, Jackson, DA, and Lipps, HJ (2007). Establishment and mitotic stability of an extra-chromosomal mammalian replicon. *BMC Cell Biol* **8**: 33.
7. Argyros, O, Wong, SP, Fedonidis, C, Tolmachov, O, Waddington, SN, Howe, SJ, *et al.* (2011). Development of S/MAR minicircles for enhanced and persistent transgene expression in the mouse liver. *J Mol Med (Berl)* **89**: 515-529.
8. Bigger, BW, Tolmachov, O, Collombet, JM, Fragkos, M, Palaszewski, I, and Coutelle, C (2001). An araC-controlled bacterial cre expression system to produce DNA minicircle vectors for nuclear and mitochondrial gene therapy. *J Biol Chem* **276**: 23018-23027.
9. Luke, J, Carnes, AE, Hodgson, CP, and Williams, JA (2009). Improved antibiotic-free DNA vaccine vectors utilizing a novel RNA based plasmid selection system. *Vaccine* **27**: 6454-6459.
10. Luke, JM, Vincent, JM, Du, SX, Gerdemann, U, Leen, AM, Whalen, RG, *et al.* (2011). Improved antibiotic-free plasmid vector design by incorporation of transient expression enhancers. *Gene Ther* **18**: 334-343.
11. Carnes, AE, Hodgson, CP, Luke, JM, Vincent, JM, and Williams, JA (2009). Plasmid DNA production combining antibiotic-free selection, inducible high yield fermentation, and novel autolytic purification. *Biotechnol Bioeng* **104**: 505-515.
12. Williams, JA, Carnes, AE, and Hodgson, CP (2009). Plasmid DNA vaccine vector design: impact on efficacy, safety and upstream production. *Biotechnol Adv* **27**: 353-370.
13. Piechaczek, C, Fetzer, C, Baiker, A, Bode, J, and Lipps, HJ (1999). A vector based on the SV40 origin of replication and chromosomal S/MARs replicates episomally in CHO cells. *Nucleic Acids Res* **27**: 426-428.
14. Noll, EM, Eisen, C, Stenzinger, A, Espinet, E, Muckenhuber, A, Klein, C, *et al.* (2016). CYP3A5 mediates basal and acquired therapy resistance in different subtypes of pancreatic ductal adenocarcinoma. *Nat Med* **22**: 278-287.

15. Hagedorn, C, Baiker, A, Postberg, J, Ehrhardt, A, and Lipps, HJ (2012). A colony-forming assay for determining the establishment efficiency of S/MAR-containing nonviral episomal expression vectors. *Cold Spring Harb Protoc* **2012**: 706-708.
16. Ansari, D, Tingstedt, B, Andersson, B, Holmquist, F, Sturesson, C, Williamsson, C*, et al.* (2016). Pancreatic cancer: yesterday, today and tomorrow. *Future Oncol* **12**: 1929-1946.
17. Rahib, L, Smith, BD, Aizenberg, R, Rosenzweig, AB, Fleshman, JM, and Matrisian, LM (2014). Projecting cancer incidence and deaths to 2030: the unexpected burden of thyroid, liver, and pancreas cancers in the United States. *Cancer Res* **74**: 2913-2921.
18. Malvezzi, M, Bertuccio, P, Rosso, T, Rota, M, Levi, F, La Vecchia, C*, et al.* (2015). European cancer mortality predictions for the year 2015: does lung cancer have the highest death rate in EU women? *Ann Oncol* **26**: 779-786.
19. Fullerton, PT, Jr., Creighton, CJ, and Matzuk, MM (2015). Insights Into SMAD4 Loss in Pancreatic Cancer From Inducible Restoration of TGF-beta Signaling. *Mol Endocrinol* **29**: 1440-1453.
20. Brandl, M, Seidler, B, Haller, F, Adamski, J, Schmid, RM, Saur, D*, et al.* (2010). IKK(alpha) controls canonical TGF(ss)-SMAD signaling to regulate genes expressing SNAIL and SLUG during EMT in panc1 cells. *J Cell Sci* **123**: 4231-4239.
21. Pardali, K, Kurisaki, A, Moren, A, ten Dijke, P, Kardassis, D, and Moustakas, A (2000). Role of Smad proteins and transcription factor Sp1 in p21(Waf1/Cip1) regulation by transforming growth factor-beta. *J Biol Chem* **275**: 29244-29256.
22. Moustakas, A, and Kardassis, D (1998). Regulation of the human p21/WAF1/Cip1 promoter in hepatic cells by functional interactions between Sp1 and Smad family members. *Proc Natl Acad Sci U S A* **95**: 6733-6738.
23. Yasutome, M, Gunn, J, and Korc, M (2005). Restoration of Smad4 in BxPC3 pancreatic cancer cells attenuates proliferation without altering angiogenesis. *Clin Exp Metastasis* **22**: 461-473.
24. David, CJ, Huang, YH, Chen, M, Su, J, Zou, Y, Bardeesy, N*, et al.* (2016). TGF-beta Tumor Suppression through a Lethal EMT. *Cell* **164**: 1015-1030.
25. Liu, CY, Lin, HH, Tang, MJ, and Wang, YK (2015). Vimentin contributes to epithelial-mesenchymal transition cancer cell mechanics by mediating cytoskeletal organization and focal adhesion maturation. *Oncotarget* **6**: 15966-15983.
26. Camara, J, and Jarai, G (2010). Epithelial-mesenchymal transition in primary human bronchial epithelial cells is Smad-dependent and enhanced by fibronectin and TNF-alpha. *Fibrogenesis Tissue Repair* **3**: 2.
27. Deer, EL, Gonzalez-Hernandez, J, Coursen, JD, Shea, JE, Ngatia, J, Scaife, CL*, et al.* (2010). Phenotype and genotype of pancreatic cancer cell lines. *Pancreas* **39**: 425-435.
28. Bode, J, Kohwi, Y, Dickinson, L, Joh, T, Klehr, D, Mielke, C*, et al.* (1992). Biological significance of unwinding capability of nuclear matrix-associating DNAs. *Science* **255**: 195-197.
29. Stehle, IM, Scinteie, MF, Baiker, A, Jenke, AC, and Lipps, HJ (2003). Exploiting a minimal system to study the epigenetic control of DNA replication: the interplay between transcription and replication. *Chromosome Res* **11**: 413-421.
30. Rupprecht, S, Hagedorn, C, Seruggia, D, Magnusson, T, Wagner, E, Ogris, M*, et al.* (2010). Controlled removal of a nonviral episomal vector from transfected cells. *Gene* **466**: 36-42.

31. Ryan, DP, Hong, TS, and Bardeesy, N (2014). Pancreatic adenocarcinoma. *N Engl J Med* **371**: 2140-2141.
32. Herreros-Villanueva, M, Hijona, E, Cosme, A, and Bujanda, L (2012). Mouse models of pancreatic cancer. *World J Gastroenterol* **18**: 1286-1294.
33. Xia, X, Wu, W, Huang, C, Cen, G, Jiang, T, Cao, J*, et al.* (2015). SMAD4 and its role in pancreatic cancer. *Tumour Biol* **36**: 111-119.
34. Kuhlmann, L, Nadler, WM, Kerner, A, Hanke, SA, Noll, EM, Eisen, C*, et al.* (2017). Identification and Validation of Novel Subtype-Specific Protein Biomarkers in Pancreatic Ductal Adenocarcinoma. *Pancreas* **46**: 311-322.
35. Yuan, W, and Varga, J (2001). Transforming growth factor-beta repression of matrix metalloproteinase-1 in dermal fibroblasts involves Smad3. *J Biol Chem* **276**: 38502-38510.
36. Edgar, R, Domrachev, M, and Lash, AE (2002). Gene Expression Omnibus: NCBI gene expression and hybridization array data repository. *Nucleic Acids Res* **30**: 207-210.
