# Supplementary material for: Novel Non-integrating DNA Nano-S/MAR Vectors Restore Gene Function in Isogenic Patient-Derived Pancreatic Tumor Models
Source: Mol Ther Methods Clin Dev. 2020 Apr 25;17:957–68. doi: 10.1016/j.omtm.2020.04.017 (PMC7218229; doi:10.1016/j.omtm.2020.04.017)
Supplement: Document S2. Article plus Supplemental Information [file mmc6.pdf]

Molecular Therapy
Methods & Clinical Development
Original Article

Novel Non-integrating DNA Nano-S/MAR Vectors Restore Gene Function in Isogenic Patient-Derived Pancreatic Tumor Models

Matthias Bozza,[1] Edward W. Green,[2] Elisa Espinet,[3] Alice De Roia,[1] Corinna Klein,[4] Vanessa Vogel,[4] Rienk Offringa,[5] James A. Williams,[6] Martin Sprick,[4] and Richard P. Harbottle[1]

[1]DNA Vector Research, German Cancer Research Center (DKFZ), Im Neuenheimer Feld 242, 69120 Heidelberg, Germany; [2]Neuroimmunology and Brain Tumor Immunology, German Cancer Research Center (DKFZ), Im Neuenheimer Feld 280, 69120 Heidelberg, Germany; [3]Division of Stem Cells and Cancer, German Cancer Research Center (DKFZ), Im Neuenheimer Feld 280, 69120 Heidelberg, Germany; [4]Stem Cells and Metastasis, Hi-Stem Heidelberg, Im Neuenheimer Feld 280, 69120 Heidelberg, Germany; [5]Molecular Oncology of Gastrointestinal Tumors, German Cancer Research Center (DKFZ), Im Neuenheimer Feld 280, 69120 Heidelberg, Germany; [6]Nature Technology Corporation, Lincoln, NE 68521, USA

We describe herein non-integrating minimally sized nano-S/MAR DNA vectors, which can be used to genetically modify dividing cells in place of integrating vectors. They represent a unique genetic tool, which avoids vector-mediated damage. Previous work has shown that DNA vectors comprising a mammalian S/MAR element can provide persistent mitotic stability over hundreds of cell divisions, resisting epigenetic silencing and thereby allowing sustained transgene expression. The composition of the original S/MAR vectors does present some inherent limitations that can provoke cellular toxicity. Herein, we present a new system, the nano-S/MAR, which drives higher transgene expression and has improved efficiency of establishment, due to the minimal impact on cellular processes and perturbation of the endogenous transcriptome. We show that these features enable the hitherto challenging genetic modification of patient-derived cells to stably restore the tumor suppressor gene SMAD4 to a patient-derived *SMAD4* knockout pancreatic cancer line. Nano-S/MAR modification does not alter the molecular or phenotypic integrity of the patient-derived cells in cell culture and xenograft mouse models. In conclusion, we show that these DNA vectors can be used to persistently modify a range of cells, providing sustained transgene expression while avoiding the risks of insertional mutagenesis and other vector-mediated toxicity.

INTRODUCTION

Rescuing the function of mutated genes in tumor cells can help to define their molecular role and provide an insight into their interactions and the processes that drive the transformation of a normal cell toward cancer without disturbing other cellular processes. A variety of different methods have been developed to generate genetically modified tumor cells, and the most effective system for delivering genes to cells is based on the use of vectors derived from modified viruses.[1] However, despite the advantages of these vectors, they also have significant limitations mainly related to their random integration into the cellular genome, the potential immunogenicity of virally encoded genes, as well as the silencing of the transgenic material over time. Each unintended consequence of genetic engineering is likely to mask or interfere with the molecular analysis of the genetic restoration. It is therefore imperative that the genetic modification has only a minimal vector-mediated impact on the molecular behavior of a cell, particularly when working with primary or patient-derived cells that are more likely to react against foreign genetic material. Previous work has established that DNA vectors comprising a nuclear scaffold/matrix attachment region (S/MAR) element and mammalian promoters allow long-term transgene expression in cancer cell lines, both *in vitro* and *in vivo*.[2–4] S/MARs mediate the binding of episomal vectors to the chromosomal scaffold during mitosis, providing sustained expression and mitotic stability over hundreds of cell divisions.[5,6] In the context of minicircle vectors, these motifs lead to a higher and more sustained transgene expression when compared to conventional plasmids, presumably due to the lack of bacterial sequences often characterized by the presence of CpG dinucleotides, responsible for the initiation of the vector silencing.[7] Nevertheless, the production of minicircles is a laborious process that implies intramolecular recombination[8] followed by purification steps designated to separate the producer vector from the minicircle. To overcome these problems, an alternative antibiotic-free selection system was established by Luke et al.[9] for the production of minimally sized plasmids. As minicircles, these antibiotic-free (AF) selectable vectors combine a highly productive fermentation (>1 g/L plasmid DNA yield) and enhanced transgene expression when compared to respective plasmids with antibiotic selection.[10–12] Herein, we describe the incorporation of the S/MAR sequence previously described by Piechaczek et al.[13] into plasmids containing an optimized bacterial backbone (pS/MAR) and minimalistic AF vectors (nS/MAR). In this study, we directly compare plasmid vectors to nanovectors and show that nS/SMAR DNA vectors

Received 31 March 2020; accepted 22 April 2020;
https://doi.org/10.1016/j.omtm.2020.04.017.

Correspondence: Richard P. Harbottle, DNA Vector Research, German Cancer Research Center (DKFZ), Im Neuenheimer Feld 242, 69120 Heidelberg, Germany.
E-mail: r.harbottle@dkfz.de

This is an open access article under the CC BY license (http://creativecommons.org/licenses/by/4.0/).

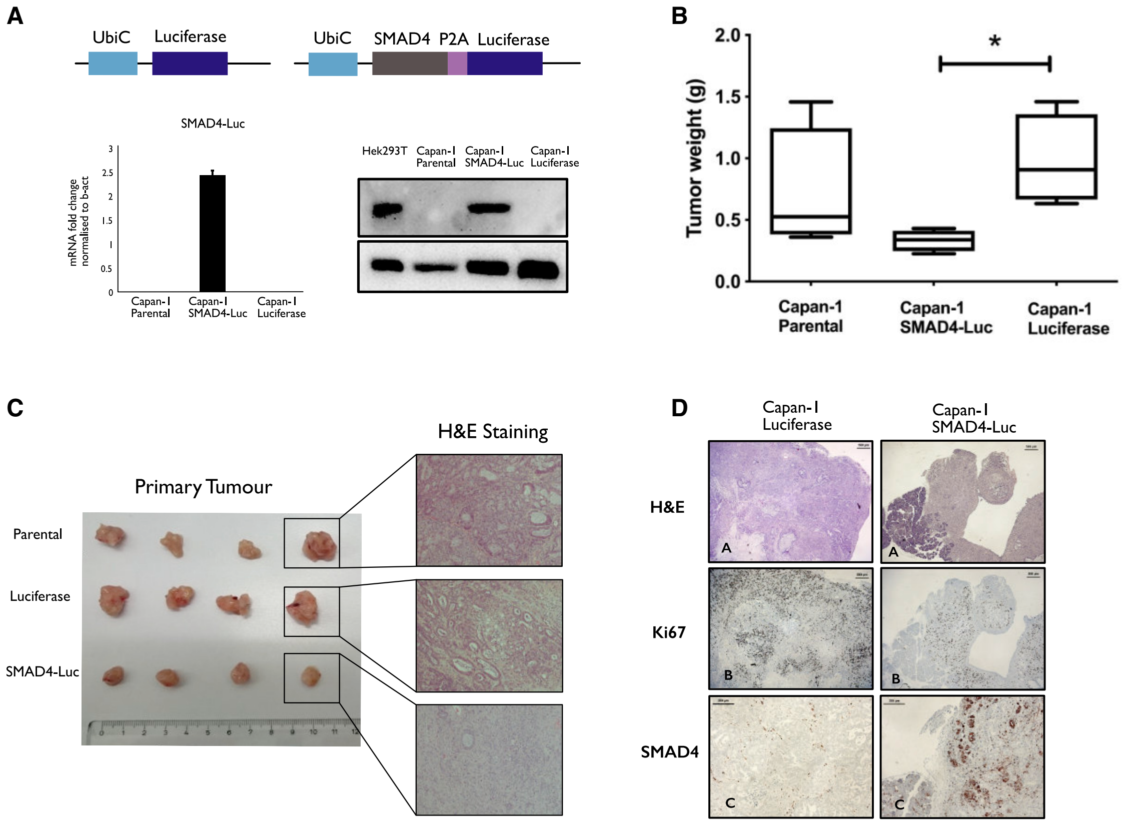

Figure 1. Delivery of pS/MAR-SMAD4 DNA Vectors Rescues the Tumorigenic Phenotype of SMAD4 Mutant Pancreatic Cancer Cell Lines

pS/MAR-luciferase (pS/MAR Luc) and pS/MAR-SMAD4-luciferase (pS/MAR SMAD4-Luc) DNA vectors were generated by introducing the transgene expression cassettes under the control of the ubiquitin C promoter (UbiC). (A) The expression of SMAD4 in modified Capan-1 was evaluated by real-time quantitative PCR (qPCR) and western blot in comparison to HEK293T cells, which constitutively express SMAD4. The impact of SMAD4 in the tumor growth was evaluated *in vivo* by injecting 5×10^5 Capan-1 cells expressing either the reporter gene luciferase or a combination of SMAD4 and luciferase orthotopically into the pancreas of NSG mice. (B) Capan-1 SMAD4-Luc cells generated significantly smaller tumors than did Capan-1 luciferase (n = 4 per group analyzed with one-way ANOVA followed by Tukey's *post hoc* test for multiple comparisons, $^*p = 0.0141$). (C) Histopathological analysis reveals that the luciferase-modified cells developed a tumor with identical morphology as those formed from the parental cell line, while the rescue of SMAD4 induces profound changes. (D) Capan-1 luciferase and Capan-1 SMAD4-Luc-derived tumors were assessed for histology by hematoxylin and eosin (H&E), proliferation (Ki67), and SMAD4 expression.

produce more robust transgene expression and have a higher efficacy in the episomal establishment of dividing cells, and we report on their application for the genetic modification of primary pancreatic cancer (PC) cells with a particular focus on vector-mediated toxicity and an analysis of the molecular integrity of the engineered cells. Herein, we report that this new class of DNA vectors has a minimal impact on the target cell genome and that they are capable of providing sustained genetic supplementation of the tumor suppressor *SMAD4* in primary pancreatic cancer models *in vitro* and *in vivo*. We conclude that this system can be considered a potent tool for the generation of reliable cancer models.

RESULTS

Generation of Capan-1 Isogenic Cells and Rescue of the Tumor Suppressor Gene *SMAD4* with Non-integrating pS/MAR Vectors

Pancreatic adenocarcinoma is one of the most lethal types of cancer,[14] with a mortality rate second only to lung cancer.[15,16] A simple and effective method to generate reliable tumor models is therefore necessary to further understand this disease. For our initial study, we used the pS/MAR DNA vector system to modify the pancreatic cancer cell line Capan-1 stably *in vitro*. We generated the pS/MAR-luciferase (pS/MAR-Luc) and the pS/MAR-SMAD4-luciferase (pS/MAR-SMAD4-Luc) vectors that were used to produce the stable cell lines Capan-1 luciferase and Capan-1 SMAD4-Luc. The tumor suppressor *SMAD4* (*DPC4* [deleted in pancreatic cancer 4]) was chosen as a model, as its loss is one of the best characterized events in pancreatic cancer development.[17] In the modified cell populations, the expression of *SMAD4* was evaluated by quantitative real-time PCR and western blot (Figure 1A), and its functional rescue was demonstrated through the activation of the SMAD4-dependent genes SnaiL[18] and p21[19,20] (Figure S1). Next, we analyzed the impact of SMAD4 restoration in *in vivo* tumor growth by injecting CAPAN-1 luciferase or CAPAN-1 SMAD4-Luc cells orthotopically into the pancreas of NSG mice. *SMAD4* expression was robustly maintained (Figure 1D),

and, as previously described,[21] its functional rescue leads to a reduction in tumor growth (Figure 1B). All mice injected with parental or luciferase control cells developed invasive primary tumors, while those injected with *SMAD4*-restored tumor cells showed small and non-invasive accumulations of transplanted cells (Figure 1C). Both DNA vectors used for the modification of these cells expressed the reporter gene luciferase, which allowed the interrogation of the presence of disseminated cells in the injected mice. Whereas control Capan-1 luciferase formed metastasis in the liver and the lungs, no metastatic events were observed in mice injected with *SMAD4*-restored cells (Figure S2A). Histopathological analysis revealed that Capan-1 luciferase cells developed tumors (Figure 1C) phenotypically similar to those formed from the unmodified parental tumor cell line, characterized by a differentiated ductal structure. In contrast, cells expressing *SMAD4* formed primary tumors that appeared less differentiated with higher recruitment of stromal cells as previously reported.[22] As the Capan-1 luciferase and parental cells generated identical primary tumors and retained a similar metastatic potential (Figure S2B), the differences observed in the tumor masses generated by Capan-1 SMAD4-Luc cells together with the restriction of their metastatic potential appear to be entirely dependent on the restoration of the tumor suppressor gene. Primary tumors from Capan-1 luciferase and Capan-1 SMAD4-Luc cell lines were compared for the phenotype (Figure 1A), proliferation with the staining of Ki67 (Figure 1B), and expression of SMAD4 (Figures 1C and 1D). Capan-1 SMAD4-Luc tumors showed a lower proliferative rate, as estimated by Ki67 expression, explaining the smaller tumor size achieved. Positive staining for *SMAD4* confirmed the DNA vector activity and capability of providing sustained transgene expression following orthotropic injection and tumor development.

Genome-wide RNA Analysis of Capan-1 Isogenic Cells

Next, we investigated the molecular changes occurring in the cells provoked by the vector and by the reintroduction of *SMAD4*. To this end, we performed genome-wide RNA profile analysis of the primary tumors formed by parental, control-luciferase, or SMAD4-Luc Capan-1 cells. The expression profiles of 351 genes were perturbed in Capan-1 luciferase cells when compared to the parental control cell (±2-fold, $p < 0.05$; Table S1; Figure 2A). Gene set enrichment analysis (GSEA) was performed to understand better the interaction between the vector and the cellular genome, which revealed enrichment for the hallmarks associated with several inflammatory responses such as a signature response to interferon-α (Figure 2C). The interferon-α pathway is associated with the cellular immune response to viral infection. It is part of the innate immunity, and it is triggered by cytoplasmic proteins that recognize an infectious agent's genome during its translocation into the nucleus. The fact that these signatures appeared enriched in the analysis suggested that pS/MAR was also recognized as a foreign entity and its presence induced an inflammatory state. The reintroduction of functional *SMAD4* was accompanied by the dysregulation of 825 genes (±2-fold, $p < 0.05$) when compared to Capan-1 parental cells. Of those, only 189 genes (Figure 2B) were specific for Capan-1 SMAD4-Luc cells when compared to Capan-1 luciferase control cells. GSEA analysis revealed strong enrichment in epithelial-to-mesenchymal transition (EMT) genes and the apoptotic hallmark signatures (Figure 2D), in line with previous reports where *ex vivo* rescue of *SMAD4* in mouse or human pancreatic cancer cells functionally induced the EMT transition that leads the apoptosis and cell death.[22] In accordance with Liu et al.[23] and Câmara et al.,[24] also in Capan-1 SMAD4-Luc cells EMT markers such as vimentin and fibronectin were shown to be upregulated in comparison to Capan-1 luciferase and parental cells (Figure S3). Although a significant number of genes were found to be perturbed in Capan-1 luciferase cells, they had limited influence on the cells' behavior during tumor development and/or the metastatic process in the experimental setting. In contrast, the interruption of cancer development, as well as the phenotypic changes observed in the Capan-1 SMAD4-Luc cells, can be attributed to the rescue of *SMAD4*.

Nano-S/MAR Is Maintained Episomally and Has an Improved Establishment Efficacy Compared to the pS/MAR Vectors

We previously showed the successful restoration of the tumor suppressor gene *SMAD4* into Capan-1 cells using the non-integrative pS/MAR DNA vector system. Although macroscopically the model reflected the results described in the literature,[25] microarray analysis showed that the plasmid vector itself had a strong impact on the transcriptome of the cells. We reasoned that these effects are a consequence of the presence of bacterial sequences in the vector, such as the origin of replication and the selection marker. These may lead to the inflammatory responses observed. To overcome this problem, we decided to introduce the S/MAR sequence derived from the human β-interferon gene cluster[26] into an optimized minimally sized antibiotic-free (AF) plasmid[9]—the nano-S/MAR vector (nS/MAR). Figure 3A represents a schematic depiction of the plasmid. The S/MAR sequence was placed after an expression cassette in which the cytomegalovirus (CMV) promoter drives the expression of the reporter gene GFP and the antibiotic selection puromycin (Puro) divided by the P2A linker sequence. The position of the S/MAR within the vector was determined by the work of Stehle et al.,[27] where they demonstrated that an active transcription upstream of the S/MAR running into this sequence was required for episomal replication and long-term vector maintenance.[28] A control vector named pS/MAR was also generated. This plasmid contained an identical expression cassette and S/MAR composition along with a bacterial backbone comprising the pUC origin of replication and a kanamycin antibiotic resistance gene. HEK293T cells were transfected with nS/MAR and pS/MAR vectors, and their capacity to establish stable cells was evaluated in a colony-forming assay as previously described[29] (Figure 3B). The nS/MAR vector generated a significantly higher ($p = 0.0003$) number of established cells that were also characterized by a significantly higher level ($p < 0.0001$) of transgene expression (Figure 3C). The molecular integrity and episomal maintenance of both nS/MAR and pS/MAR vectors in the established cell populations were determined 35 days after DNA delivery by Southern blot (Figure 3D) where DNA isolated from the modified cells was compared to linearized control vectors. The absence of smears or alternative bands to those that matched the size of the control vectors confirms

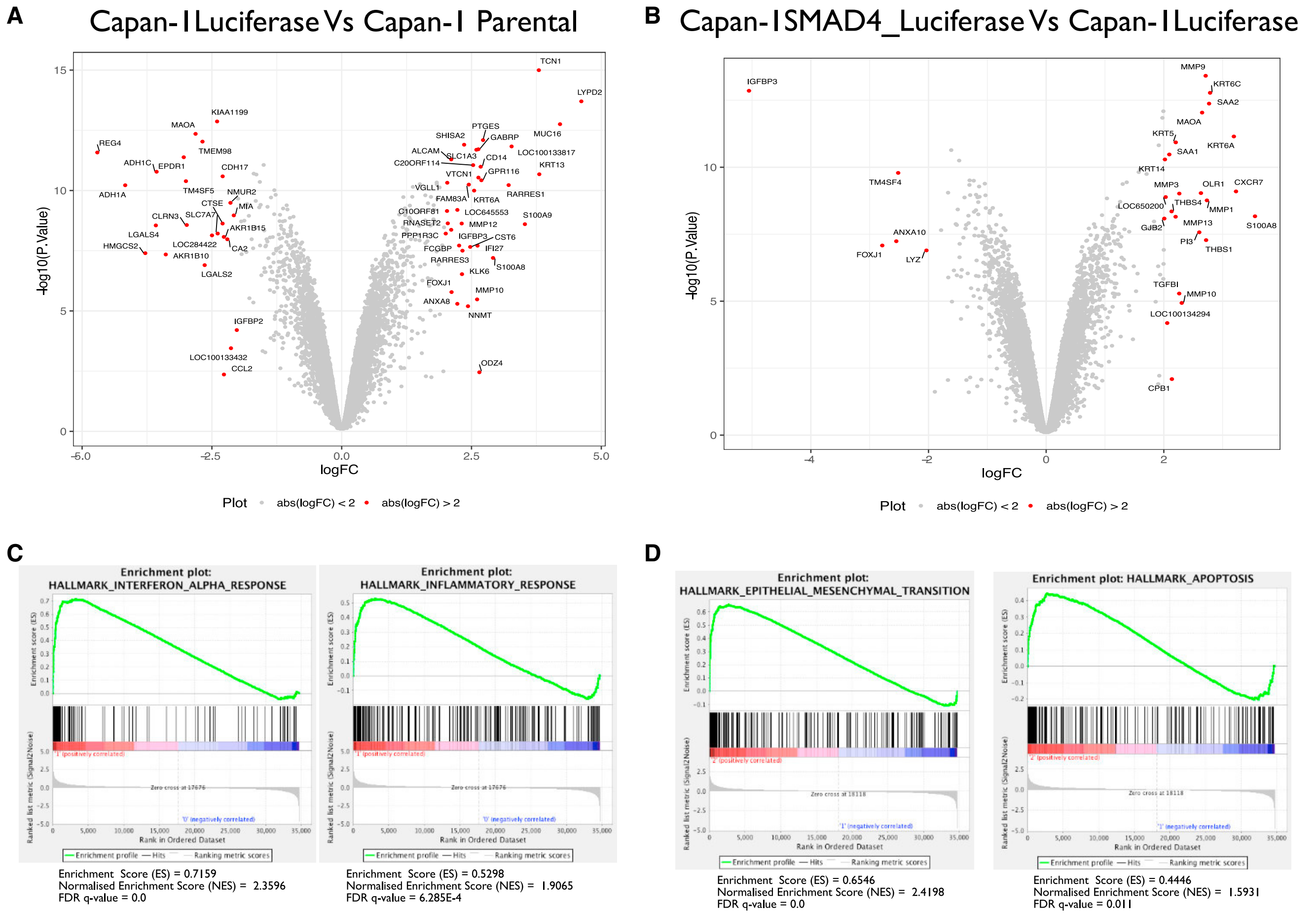

Figure 2. Genome-wide Transcriptome Analysis of modified Capan-1 cells

(A and B) Volcano plots showing the gene expression changes of Capan-1 luciferase versus Capan-1 parental (A) and Capan-1 SMAD4-luciferase versus Capan-1 luciferase (B). Highlighted in red are genes with a fold change of 2 and $p < 0.05$. (C) Gene set enrichment analyses (GSEA) of interferon-α and inflammatory response in Capan-1 luciferase versus parental cells. (D) GSEA of epithelial-to-mesenchymal transition and apoptosis signature in Capan-1 SMAD4 versus Capan-1 luciferase cells. ES, enrichment score; NES, normalized enrichment score; FDR, false discovery rate.

the stable maintenance of the plasmids as well as their extrachromosomal replication and transmission during cell division.

Genetic Modification of Primary Pancreatic Cancer Cells with Nano-S/MAR Vectors and Rescue of *SMAD4* Functionality

To evaluate the efficacy of the new nS/MAR vectors, we utilized a well-described and representative patient-derived xenografted (PDX) pancreatic cancer cell line.[30] These cells, named PACO2, were derived from an aggressive pancreatic ductal adenocarcinoma by the orthotopic expansion of a patient's tumor biopsy in NSG mice and further isolation and culture of the epithelial tumor cells. The cells have been maintained at low passage in carefully controlled media and have been characterized to closely represent the primary tumor cells from which they are derived.[30] These cells provide an ideal platform to test the nS/MAR system because of their chronic, high levels of anti-pathogenic cytokines such as interferon that make them a challenge to transfect with typical plasmids, which carry a traditional bacterial backbone. PACO2 cells carry a mutation in their *SMAD4* locus, which allowed us to test the rescue of this tumor suppressor gene in these PDX cells.

First, both pS/MAR-GFP and nS/MAR-GFP DNA vectors were transfected into PACO2 cells. The transfection efficiency and the viability of the cells were then assessed by fluorescence-activated cell sorting (FACS) (Figure 4A). Strikingly, the nS/MAR-GFP vector performed significantly better than the pS/MAR-GFP plasmids, demonstrating that these vectors can be efficiently delivered to primary human cells, which are typically refractory to transfection. The cells also retained high viability compared to cells transfected with the pS/MAR DNA vector throughout this procedure. Using the nS/MAR technology, we then generated two different engineered PACO2 cell lines: PACO2 nS/MAR-GFP (PACO-2 GFP) cells, which express the reporter gene, and PACO2 nS/MAR-SMAD4-GFP (PACO-2 SMAD4-GFP) cells, in which the expression of *SMAD4* is coupled

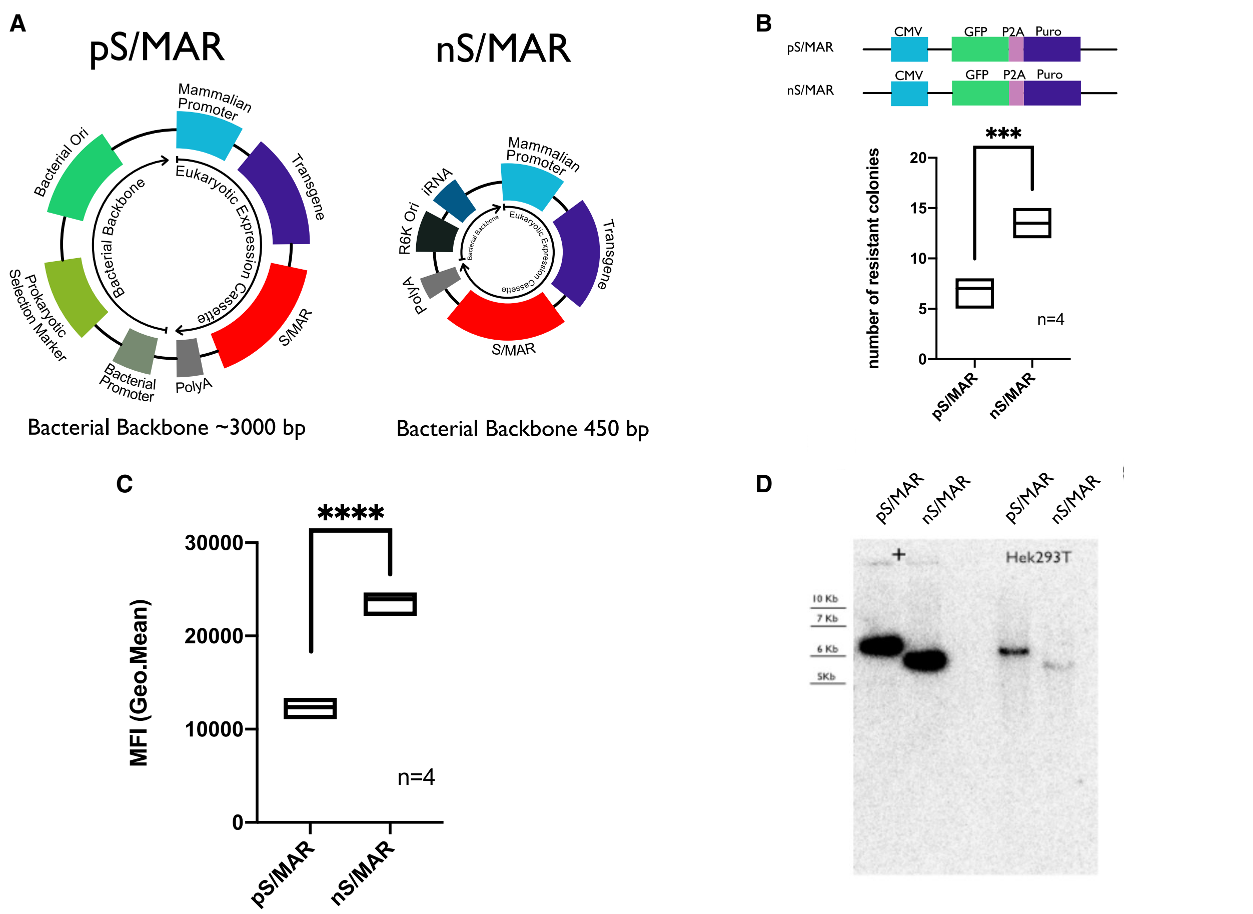

Figure 3. Nano-S/MAR Has Improved Establishment Efficiency, Sustains Higher Transgene Expression, and Is Maintained Episomally in the Nuclei of Target Cells

(A) Schematic depiction of pS/MAR and nS/MAR DNA vectors. In nS/MAR vectors, the RNA-OUT R6K Ori system replaces the bacterial backbone comprising the bacterial origin of replication and the selection marker. (B) Number of colonies 35 days after GFP$^+$ selection. In the plots the line represents the median of four independent replicates per group analyzed with the t test for significance (p = 0.0003). (C) nS/MAR vectors were shown to provide a more robust transgene expression in established cells when compared to the respective plasmid with the bacterial backbone (analyzed by t test $p < 0.0001$). (D) Southern blot showing the molecular integrity and the episomal maintenance of the vectors.

via a P2A sequence to the GFP. After 30 days the modified cells were analyzed by flow cytometry for the expression of the reporter gene GFP (Figure 4B), which showed that the PACO2 GFP cells produced high levels of transgene expression (Figure 4C). The reintroduction of *SMAD4* was confirmed by western blot (Figure 4C) using Panc-1, a pancreatic cancer cell line with no mutation or loss of the *SMAD4* genetic locus, as a control reference. The molecular integrity and episomal stability of the nanovectors in modified primary cells were determined via Southern blot (Figure S4) where the presence of single sharp bands of the expected size confirmed the extrachromosomal maintenance and episomal segregation of these plasmids in dividing cells.

Impact of nS/MAR Vector in the Transcriptome of Modified Cells

We then investigated the impact of the nanovectors on the PDX cells and the molecular and cellular consequences caused by the restoration of *SMAD4.* For this, genome-wide RNA profile analysis of the established GFP and SMAD4-GFP PACO2 cells, as well as the parental lines, were performed. Four biological replicates were prepared for each cell line 1 month after DNA delivery, and the differences in the relative gene expression profiles of each were evaluated. Strikingly, the nS/MAR-GFP vector had a minimal impact on the cells (Figure 4D) with only two genes having appeared significantly perturbed ±2-fold ($p < 0.05$; Table S3). In contrast, the re-introduction of functional *SMAD4* generated a more prominent effect on the cellular transcriptome, with 169 genes that appeared perturbed when the same analysis was performed (Figure 5A; Table S4).

GSEA was then performed to prove that the vectors can provide the sustained expression of functional *SMAD4 in vitro.* The EMT and the transforming growth factor β (TGF-β) pathways (Figure 5B) were

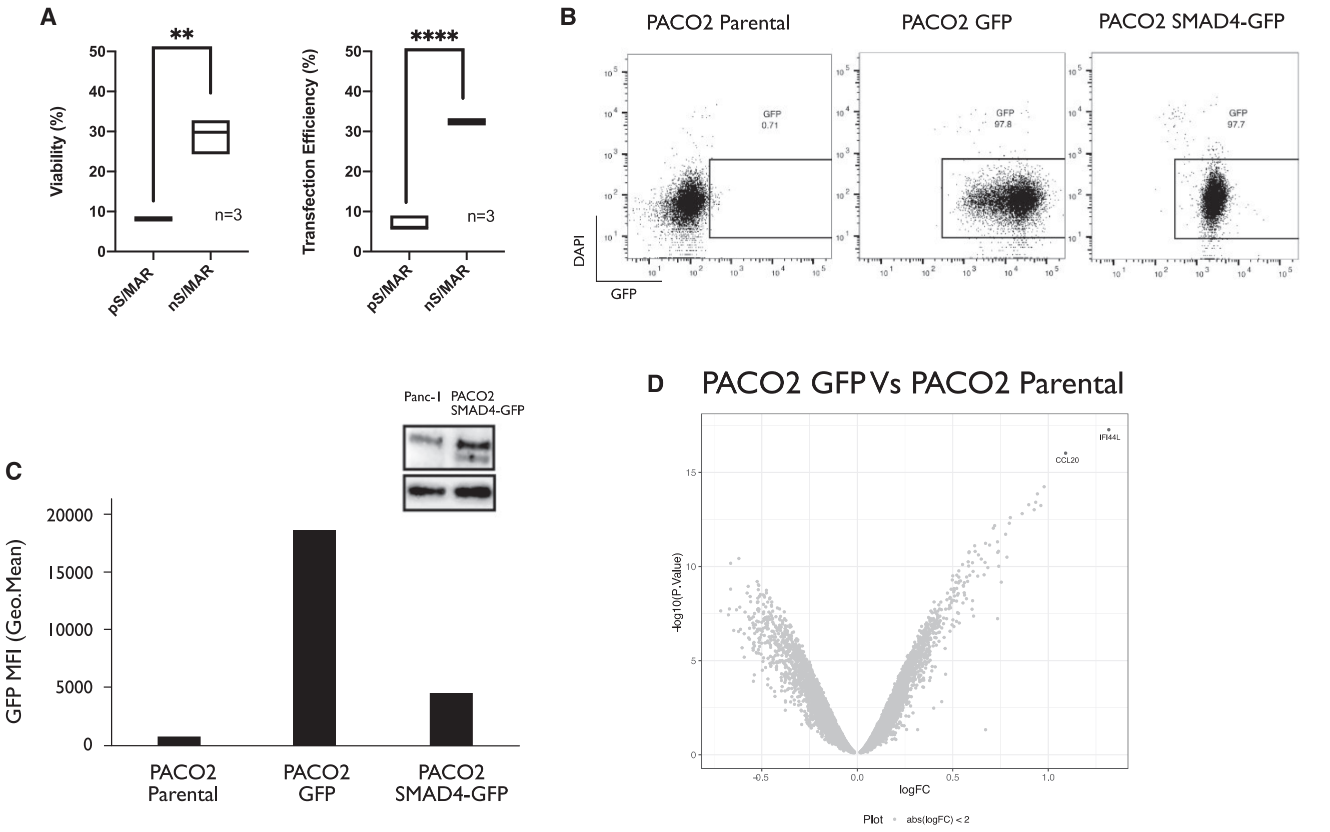

Figure 4. Genetic Modification of Primary Pancreatic Tumor Cells (PACO2) Using nS/MAR DNA Vector Technology

pS/MAR and nS/MAR vectors expressing the reporter gene GFP were transferred into PACO2 cells through electroporation. (A) The viability and the efficiency (number of viable GFP$^+$ cells) of transfection were evaluated by flow cytometry and are represented as boxplots, where the line represents the median (n = 3, analyzed by t test; viability, p = 0.0012; transfection efficiency, p < 0.0001). (B) PACO2 primary pancreatic cancer cells were established with nS/MAR-GFP and n/MAR-SMAD4-GFP (nS/MAR-SMAD4). (C) The expression of the reporter gene GFP was evaluated in flow cytometry in comparison to parental unmodified PACO2 cells. Western blot analysis shows successful re-introduction of SMAD4 in nS/MAR-SMAD4 PACO2 cells. SMAD4 wild-type (WT) Panc-1 cells are shown as a control. (D) Volcano plot showing the gene expression changes of PACO2 GFP versus parental cells. Highlighted in red are genes with a fold change of 2 and p < 0.05.

enriched, demonstrating the capability of the nS/MAR vector technology to provide genetic rescue and the sustained and restored functionality of the tumor suppressor gene *SMAD4*.

Modified Primary Pancreatic Cancer Cell Xenograft Models and *SMAD4* Rescue

Finally, to evaluate the *in vivo* impact of the nanovectors on the behavior of PACO2 cells, 0.5×10^5 cells were injected orthotopically into NSG mice. PACO2-GFP, PACO2 SMAD4-GFP, and the PACO2 parental cells were used for the study, and the xenografted tumors in the pancreas were evaluated for morphology, proliferation, and *SMAD4* expression.

All (12/12) mice injected with PACO2 GFP developed primary tumors in their pancreases, and in 11/12 cases the tumor also grew out into the abdominal cavity. Additionally, every (10/10) mouse treated with PACO2 parental cells formed tumors in their pancreas, and 4/10 also developed masses in the abdominal cavity. This markedly contrasts with only 2/12 mice injected with PACO2 SMAD4-GFP developing primary tumors in their pancreas, and none showing cells outgrowing into their abdominal cavity (Figure S5). The total weight of the pancreases was used as a measure of tumor growth, and mice injected with the *SMAD4*-restored cells had significantly smaller tumors than did mice injected with the unmodified parental line and GFP cell line (Figure 5C). Tumors formed by PACO2 GFP cells presented similar morphology to those created with the unmodified parental PACO2 cells (Figure 5D) with tumorous ductal glands and extensive fibrosis. They showed active proliferation and were characterized by the absence of *SMAD4* expression. Histological analysis of the few tumors that grew in the pancreas of mice injected with PACO2 SMAD4-GFP cells revealed that these tumors were derived from most likely cells that do not express *SMAD4* and they were actively proliferating (Ki67 positive) (Figure 5E). Moreover, their morphology matched those of PACO2 GFP and PACO2 parental cells. PACO2 cells modified with the reporter gene maintained their original behavior, demonstrating that the episomal nS/MAR vector

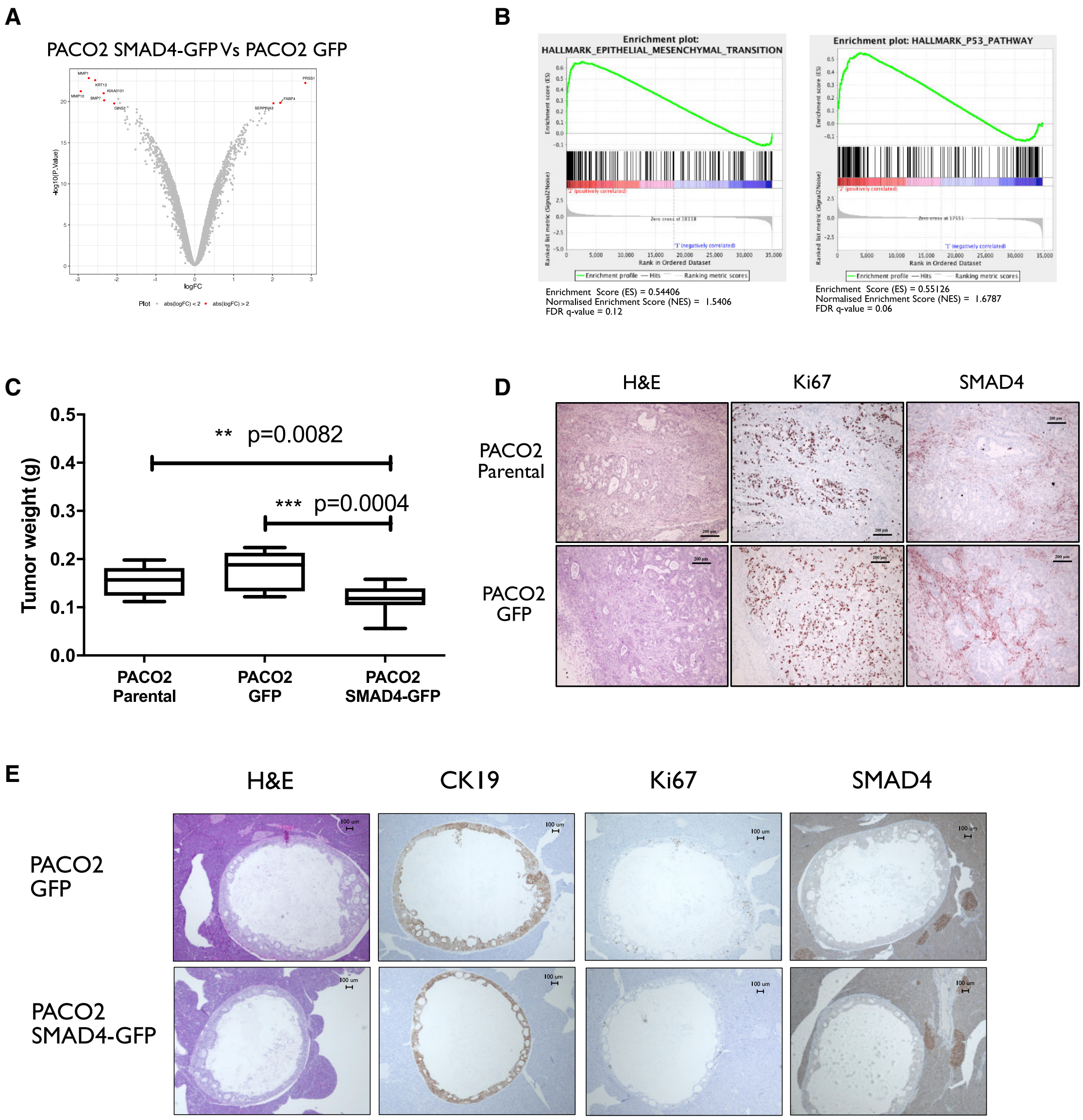

Figure 5. *In Vitro* Restoration of SMAD4 Functionality in Primary Models

(A and B) Differentially expressed genes were analyzed with a >2-fold and less than −2-fold log fold change (FC) ($p < 0.05$) (A) and further validated in gene set enrichment analysis (GSEA) (B), where PACO2 SMAD4 cells showed strong enrichment for the hallmarks underlying the epithelial-to-mesenchymal transition and the activation of the TGF-β pathways when compared to the PACO2 GFP control line. (C) The impact of SMAD4 on tumor growth was evaluated *in vivo* by injecting 5×10^5 PACO2 parental, PACO2 GPF, and PACO2 SMAD4 cells orthotopically into the pancreas of NSG mice. The weight of the pancreas was used as a measure of tumor growth, and we showed that mice injected with PACO2 cells had significantly lighter organs than did animals injected with the control cell lines (n = 10 for PACO2 parental, n = 12 for PACO2 GFP and SMAD4 analyzed by one-way ANOVA followed by the Tukey's *post hoc* test for multiple comparisons). (D) Primary tumors obtained from the orthotopic injection of not modified and PACO2 GFP cells were assessed for morphology with H&E staining, proliferation via staining with the proliferative marker Ki67, and for the expression of SMAD4. The tumors formed from PACO2 modified with the reporter gene GFP showed a defined ductal differentiation typical of pancreatic cancers with a high proliferate rate and negative expression for SMAD4 identical to unmodified parental PACO2 cells. (E) Cells where SMAD4 functionality was restored did not form tumors when engrafted into mice. The outgrowing masses originated from human cells (CK19 positive), were actively proliferating (Ki67 positive), and did not stain positive for SMAD4 expression.

had no effect on the behavior of the cells or their molecular integrity. Therefore, the observed impact on the *SMAD4* rescued cells can be fully attributed to the restored functionality of the tumor suppressor gene.

DISCUSSION

Pancreatic ductal adenocarcinoma (PDA) is considered one of the most malignant types of cancer with relatively late detection and poor prognosis,[14] with only 10%–15% of patients eligible for surgery,

which is currently the only curative option.[31] Several methods have been developed to unravel the genetic basis of PDA ranging from the generation of transgenic mouse models[32] to the *in vitro* modification of cancer cell lines and explanted primary cells. The *in vitro* or *ex vivo* genetic modification of cells relies on the use of integrating vectors to generate cells that can persistently and stably express a gene of interest through cell division. These systems are mostly represented by integrating viral vectors such as lentivirus that take advantage of the integrating nature of the virus to transfer the genetic material into the cellular genome. In this process, the integration of the gene of interest happens randomly into the target cells, which may result in unstable transgene expression over time. The necessity of adequate facilities for viral handling as well as the laborious process necessary for their preparation and purification make these vectors not the ideal tool to swiftly generate tumor models. Then, viral antigens and nucleic acids can trigger the cellular immune response against infectious agents that generate undesired perturbation in the cellular and molecular behavior, leading to a high background during the studies. S/MAR vectors are a unique class of DNA constructs that can provide long-term transgene expression and mitotic stability in mammalian cells without relying on toxic viral components or random integration that can potentially disrupt the molecular behavior of the targeted cells. In this study, the pS/MAR vector was engineered with the aim of generating pancreatic cancer tumor models with a particular application of the rescue of the tumor suppressor gene *SMAD4*. The loss of *SMAD4* is one of the best characterized events in pancreatic cancer development. In approximately 30% of all pancreatic cancers, *SMAD4* is homozygously deleted, and another 20% display missense, nonsense, or frameshift mutations. The downregulation of *SMAD4* counteracts TGF-β-induced cell cycle arrest and apoptosis, but the restoration of this tumor suppressor gene can reverse the invasive phenotype as well as attenuate the proliferation of pancreatic cancer cells.[33] In this study, we generated two vectors: pS/MAR-Luc, responsible for the expression of the reporter gene luciferase, and pS/MAR SMAD4-Luc, where the expression of the tumor suppressor gene *SMAD4* was linked to luciferase through the P2A self-cleavage sequence. These vectors were used to engineer the commonly used pancreatic cancer cell line Capan-1 genetically. We first investigated the capacity of the vectors to generate stable cell lines and to restore the expression of *SMAD4 in vitro* before these cells were used for the generation of orthotropic xenograft mouse models. We showed not only that we could restore the expression of a key tumor suppressor gene *in vitro*, but also that the engineered cells retained a stable expression of the transgene *in vivo*. The histopathological analysis of Capan-1 luciferase tumors showed that the modification of the cells with this vector technology had a minimal impact on the cells' behavior, as they formed tumors that displayed a highly differentiated pancreatic adenocarcinoma morphology similarly to tumors formed from the unmodified parental Capan-1 cell line. The metastasis in the liver and in the lungs of mice injected with parental Capan-1 and Capan-1 luciferase cells also confirmed that the presence of the vector did not molecularly alter the cells' behavior, and they retained their aggressive metastatic potential. In contrast, Capan-1 SMAD4-Luc cells formed tumors in the pancreas that appeared smaller with a lost capability of forming metastasis. Although the presence of the episomal vector did not influence the growth of the cells, the transcriptome analysis of Capan-1 luciferase cells revealed that at the molecular level the presence and the extrachromosomal replication of plasmids carrying bacterial sequences were responsible for the perturbation of about 400 genes that are mostly associated with antiviral and inflammation responses. These findings are in accordance with previous reports, where it was demonstrated that the presence of bacterial sequences in plasmids is responsible for the cell responses against foreign DNA that leads to its epigenetic silencing. As expected, the transcriptome analysis did not show upregulation for the transcripts responsible for the expression of the *SMAD4* protein, as the microarray probes do not match the codon-optimized transgenic sequence (Figure S6). To improve the efficacy of our DNA vector technology and to test the potential toxicity of extraneous bacterial sequences, we decided to swap the bacterial backbone composed of a pUC origin of replication and the selectable selection kanamycin for the RNA-OUT system developed at Nature Technology Corporation (NTC). We called this novel class of minimally sized antibiotic-free vector DNA plasmids nano-S/MAR (nS/MAR). This new generation of DNA vectors was compared to the respective traditional plasmids in HEK293T cells, and we could show that the nS/MAR vectors had a higher establishment of efficacy and that they sustained more robust transgene expression in the established clones over time for a minimum of 35 days. In accordance with what was previously reported by Argyros et al.,[3] cells that are modified with a plasmid carrying minimal bacterial sequences are characterized by a higher transgene expression, most likely due to a significant reduction in the CpG content. Also, in this novel class of vectors, the presence of the S/MAR sequence mediated the extrachromosomal replication, as we could demonstrate by Southern blot. We further challenged this vector technology in human primary pancreatic cells (PACO2). PACO2 cells were demonstrated to have a high constant secretion of the antiviral cytokine interferon-β,[34] which makes them challenging to transfect with a canonical plasmid. The benefits of having a minimally sized bacterial backbone were evident in PACO2 cells where nS/MAR vectors could be more effectively delivered to cells. Furthermore, for the first time, we showed that the nS/MAR vectors could successfully be established in primary cells *ex vivo* and that the portion of unmodified cells was substantially reduced. As PACO2 cells carry a mutation in the *SMAD4* locus, we could demonstrate the successful establishment of cell lines where we rescued the functionality of the tumor suppressor gene with nS/MAR vectors. At the molecular level, we investigated the impact of nS/MAR vectors on PACO2 cells by measuring their genome-wide RNA expression levels. PACO2-GFP cells surprisingly showed that although they had undergone the selection process and they were grown for 30 days with the episomal vectors, only two genes were perturbed by the process with changes in their expression profiles. This result shows the minimal impact of the vector system in primary cells during the engineering process. In high contrast, the PACO2 cells in which the expression of *SMAD4* was rescued showed more profound changes, particularly in genes that were previously reported to be associated with *SMAD4* functionality such as the transcriptional regulation of MMP1.[35] The enrichment in the signature associated with TGF-β activation and EMT demonstrated the activity of the tumor suppressor gene and, indirectly, that the nS/

MAR technology can provide the sustained expression of functional *SMAD4* throughout hundreds of cell divisions. The parental and GFP PACO2 cells engrafted in mice with equivalent efficiency, forming indistinguishable tumors that grew out from the pancreas and into the abdominal cavity. The immunohistochemical analysis showed that the genetic modification of these cells with an nS/MAR-GFP had no impact on the behavior of the cells. They formed tumors that displayed the same aggressive phenotype characterized by ductal structures and active proliferation as demonstrated with the Ki67 staining. In contrast, the restoration of *SMAD4* induced profound changes. The injected cells did not develop tumors in most mice, and in these cases where an outgrown tumor was found, it did not show the expression of the tumor suppressor, indicating that it arose from escaper cells. Before injection of PACO2 SMAD4-GFP cells, FACS analysis revealed that ~97% of the cells were positive for the expression of the transgenic construct, and it is likely that the negative fraction of modified cell populations induced the tumor engraftment in a few mice.

In this study, we conclude that although the originally described pS/MAR vectors can, in principle, be used to genetically modify most cell types, the presence of their large bacterial backbone represents a limitation in the application of this vector technology to primary cells that are known to respond against foreign DNA sequences. Herein, we show the generation of the nS/MAR nanovector platform, and we demonstrate its enhanced efficiency at modifying and generating novel cell lines that are characterized by higher transgene expression and lower vector-mediated molecular perturbation. We think that the novel nS/MAR DNA vector system will prove to be a valuable genetic tool useful for the generation of persistently modified isogenic cells, providing the utility to evaluate the expression of transgenes with minimal vector-mediated impact in cultured cell lines or typically refractory primary and patient-derived cells.

MATERIALS AND METHODS

Vector Construction

All of the vector modifications on pS/MAR were performed using the InFusion cloning strategy (Takara Biotech) following the manufacturer's guidelines.

The vector pS/MAR ubiquitin C (UbiC)-luciferase was generated from the original pEPI vector. The UbiC promoter was introduced into the plasmid at the PcI restriction site (pS/MAR-UbiC), and the luciferase transgene was subsequently cloned into the vector through the BglII site. The luciferase-p2a-SMAD4 expression cassette was generated via PCR and cloned into the pS/MAR-UbiC plasmid at the BglII cloning site. p/MAR GFP was created replacing the GFP expression cassette of the original pEPI plasmid with the GFP-p2a-puromycin cassette generated by PCR.

nS/MAR-GFP and nS/MAR-SMAD4 were generated by swapping the bacterial backbone of the respective canonical plasmids with the R6K-RNA-OUT system developed at Nature Technology Corporation.

Cell Culture Conditions and Transfection

HEK293T cells were maintained in DMEM medium (Sigma) supplemented with 10% fetal bovine serum (FBS) (Gibco) and 1× penicillin/streptomycin (Sigma). DNA constructs were transfected into HEK293T cells using JetPEI DNA transfection reagent (Polyplus-transfection). PACO2 cells were cultured as described by Noll et al.,[30] and the transfection was carried with the Amaxa 4D-Nucleofector (Lonza) following the guidelines by Lonza. Briefly, 1×10^6 cells prior to transfection were isolated with Accutase (PromoCell) treatment and centrifuged at $200 \times g$ for 5 min at room temperature. The supernatant was discarded, and the cells were re-suspended carefully in 100 μL of room temperature supplemented with Nucleofector solution SF per sample. 2 μg of plasmid DNA was then added to the solution, and the tube was gently flanked. The transfection was achieved by applying the pulse CM-120. After the pulse, 500 μL of pre-warmed media was added to the cuvette, and the cells transferred into a new well of a 12-well plate containing 1 mL of pre-warmed growth medium.

Colony-Forming Assay

HEK293T cells were transfected with pS/MAR and nS/MAR. For each construct, 24 h post-transfection 100 positively transfected cells were plated into a 6-cm tissue culture dish after FACS sorting (FACSAria II) and cultured in the presence of 0.5 μg/mL puromycin (PanReac AppliChem) for 3 days. Cells were then cultured for 4 weeks in the absence of selection. Resistant colonies were fixed with 1% formaldehyde/PBS for 15 min at room temperature and subsequently stained with 0.5% crystal violet/25% methanol for 10 min at room temperature, as previously reported.[29] Afterward, plates were rinsed with double distilled H_2O (ddH_2O) to remove excessive staining solution, and the colonies were counted.

Statistical Analysis

The results were generated using biological and technical replicates throughout each experiment. For data analysis, an unpaired t test was performed where the comparison was restricted to two groups, whereas when the analysis was extended to three or more groups, a one-way ANOVA followed by a Tukey's post hoc test was performed for multiple comparisons.

Quantitative Real-Time PCR

Total RNA from Capan-1 parental, luciferase, and SMAD4 cells was extracted with the RNeasy kit (QIAGEN) and treated with a DNA-free kit (Thermo Fisher Scientific) following the manufacturers' guidelines. 1μg of RNA was then reverse transcribed to cDNA with the RevertAid first strand cDNA synthesis kit (Thermo Fisher Scientific) prior to real-time quantitative PCR (qPCR) analysis into the LightCycler 96 (Roche). The expression of the transgenic construct SMAD4-P2A-luciferase (forward, 5′-ATCGGCAGCGGCGC-3′, reverse, 5′-GGGCCCAGGGTTTTCC-3′) was analyzed in Capan-1 parental, luciferase, and SMAD4-luciferase cells in comparison to the housekeeping gene β-actin (forward, 5′-CCTCGCCTTTGCTG CCGATCC-3′, reverse, 5′-GGATCTTCATGAGGTAGTCAGTC-3′). The following primers pairs were used to perform real-time

quantitative PCR (qPCR) to evaluate the expression of vimentin (forward, 5′-TACAGGAAGCTGCTGGAAGG-3′, reverse, 5′-ACCAG AGGGAGTGAATCCAG-3′), SnaiL (forward, 5′-GCTGCAGGA CTCTAATCCAGA-3′, reverse, 5′-ATCTCCGGAGGTGGGATG-3′), p21 (forward, 5′-TGAGCCGCGACTGTGATG-3′, reverse, 5′-GTC TCGGTGACAAAGTCGAAGTT-3′), and fibronectin (forward, 5′-GGGAGAATAAGCTGTACCATCG-3′, reverse, 5′-TCCATTA CCAAGACACACACACT-3′).

Western Blot Analysis

Cells were lysed in ice-cold radioimmunoprecipitation assay (RIPA) buffer (Cell Signaling Technology) for 30 min on ice, then the lysate was centrifuged at 12,000 × *g* for 15 min at 4°C, and the supernatant was transferred into a new tube. The proteins were separated by 4%–15% gradient SDS-PAGE and then transferred to polyvinylidene fluoride (PVDF) membranes using the standard program P3 (20 V for 7 min) of the iBlot 2 (Life Technologies). Western blot analyses were performed with the primary antibody anti-SMAD4 (B8, Santa Cruz Biotechnology), anti-p21 (C19, Santa Cruz Biotechnology), anti-SnaiL (L70G2, Cell Signaling Technology), anti-α-tubulin (DM1A, Sigma-Aldrich), and anti-GAPDH (G9, Santa Cruz Biotechnology). The peroxidase AffiniPure goat anti-mouse immunoglobulin G (IgG) (Jackson ImmunoResearch) was used as a secondary antibody to resolve the blots.

Southern Blot Analysis

For DNA analysis, total DNA was extracted using the DNA Blood & Tissue kit (QIAGEN) and quantified using a NanoDrop 2000c spectrophotometer (Thermo Fisher Scientific). For Southern blot analysis, total DNA (10–15 μg) was digested overnight with BamHI mixed with 10× loading dye and separated on an 0.8% agarose gel at 20 mV overnight. The gel was immersed in 0.25 M HCl for 10 min, incubated twice for 15 min in depurination buffer, followed by a 15-min incubation in neutralization buffer. The gel was supported on a layer of Whatman 3MM paper with a tank containing 20× saline sodium citrate (SSC) nucleic acid transfer buffer. A Hybond-XL nylon membrane from Amersham Biosciences was soaked with buffer and placed on top of the gel, taking care to remove any bubbles. Once the paper towel was positioned, a weight was balanced on top, and the apparatus was left overnight to allow the complete transfer. The following day the apparatus was disassembled, and the nylon membrane was exposed to UV radiation for 1 min to cross-link the DNA to the membrane permanently. The GFP gene was used to generate DNA fragments that were labeled with 32P (Prime-It II random primer labeling kit, Agilent Technologies) and used as a probe. The hybridization was performed in Church's buffer at 65°C for 16 h.

FACS Analysis

For FACS analysis, HEK293T and PACO2 cells were detached from their culturing vessels with either trypsin or Accutase, washed three times in cold PBS, and resuspended in PBS containing 1% FBS. Prior to flow cytometry analysis (LSRFortessa, Becton Dickinson), the viability staining was performed by adding DAPI or the 7-aminoactinomycin D (7AAD) live/dead marker. Analysis of data was performed with the FlowJo software, which was also used to measure the median fluorescence intensity of the populations established with vector expression for the reporter gene GFP.

Orthotopic Injection

NOD.*Prkdc^{scid}.Il2rg^{null}* (NSG) mice were bred and housed under specific pathogen-free conditions at the central animal facility of the German Cancer Research Center (DKFZ). Female mice were used for the studies. All animal experiments were approved by the Governmental Committee for Animal Experimentation (Regierungspräsidium Karlsruhe).

For the orthotopic tumor growth experiments, 200,000 cells were mixed with Matrigel (2 mg/mL; Becton Dickinson) and injected into the mice's pancreas. Engraftment of tumors and subsequent growth were monitored by regular palpation of the implantation site.

Gene Expression Analyses

For the gene expression analysis of modified Capan-1, xenografted cells were harvested from the pancreas of the injected mice, and the RNA was extracted with the RNAeasy kit (QIAGEN), generating four replicates for each condition. The RNA from modified PACO2 cells was extracted with the same methodology. The DKFZ Core Facilities team performed expression analysis experiments using Illumina HumanHT-12 v4.0 gene expression BeadChips. The top differentially expressed genes were calculated using the limma package in RStudio employing an empirical Bayes model to generate moderated t test results. Figures were generated using the ggplot2 package. GSEA was conducted using the GSEA desktop application and the gene sets downloaded from the Broad Institute with 1,000 permutations. Quantile-normalized expression data were used as input.

Immunohistochemistry

Tumor specimens were fixed in 10% formalin overnight and embedded in paraffin. For immunohistochemistry, slides were deparaffinized and rehydrated. Antigen retrieval was enhanced by boiling in a steam pot at pH 6 in Dako target retrieval solution (Dako) for 15 min, followed by cooling for 30 min and washing in distilled water. Nonspecific binding was blocked by using the Linaris avidin/biotin blocking kit (Vector Laboratories) according to the manufacturer's instructions. Slides were incubated with primary antibodies for 30 min, rinsed in PBS-T (PBS with 0.5% Tween 20), incubated for 20 min with the appropriate secondary antibody using the Dako REAL detection system (Dako), and rinsed in PBS-T. After blocking of endogenous peroxidase and incubation with streptavidin-horseradish peroxidase (HRP) (20 min at room temperature), slides were developed with 3-amino-9-ethylcarbazole (AEC) (Dako) and counterstained with hematoxylin. All antibodies were diluted in Dako antibody diluent, including anti-SMAD4 (dilution 1:50; Santa Cruz), Ki67 (dilution 1:1,000, Sigma), and CK19 (dilution 1:200, Abcam).

Data Availability

The data discussed in this publication have been deposited in NCBI's Gene Expression Omnibus[36] and are accessible through GEO: GSE142115 and GSE142117.

SUPPLEMENTAL INFORMATION

Supplemental Information can be found online at https://doi.org/10.1016/j.omtm.2020.04.017.

AUTHOR CONTRIBUTIONS

M.B. planned and performed the experiments, analyzed the data, and wrote the manuscript; E.W.G. performed the microarray analysis; A.D.R. participated in the experiments; C.K. performed the orthotopic injections; V.V. performed the immunohistological staining; E.E. planned the experiments and wrote the manuscript; R.O. and M.S. supervised the project; J.A.W. produced the nanoplasmids; and R.P.H. planned the experiments and wrote the manuscript.

CONFLICTS OF INTEREST

J.A.W. has commercial interests in Nature Technology Corporation (Lincoln, NE, USA).

ACKNOWLEDGMENTS

We thank the microarray unit of the DKFZ Genomics and Proteomics Core Facility for providing the Illumina whole-genome expression BeadChips and related services. This work was supported by the German Cancer Research Center. The graphical abstract was generated from graphics modified from Servier Medical Art by Servier licensed under a Creative Commons Attribution 3.0 Unsupported License, Les Laboratoires Servier (www.servier.com).

REFERENCES

1. Dinca, E.B., Sarkaria, J.N., Schroeder, M.A., Carlson, B.L., Voicu, R., Gupta, N., Berger, M.S., and James, C.D. (2007). Bioluminescence monitoring of intracranial glioblastoma xenograft: response to primary and salvage temozolomide therapy. J. Neurosurg. *107*, 610–616.

2. Argyros, O., Wong, S.P., Niceta, M., Waddington, S.N., Howe, S.J., Coutelle, C., Miller, A.D., and Harbottle, R.P. (2008). Persistent episomal transgene expression in liver following delivery of a scaffold/matrix attachment region containing non-viral vector. Gene Ther. *15*, 1593–1605.

3. Argyros, O., Wong, S.P., Gowers, K., and Harbottle, R.P. (2012). Genetic modification of cancer cells using non-viral, episomal S/MAR vectors for in vivo tumour modelling. PLoS ONE *7*, e47920.

4. Wong, S.P., Argyros, O., Coutelle, C., and Harbottle, R.P. (2011). Non-viral S/MAR vectors replicate episomally in vivo when provided with a selective advantage. Gene Ther. *18*, 82–87.

5. Argyros, O., Wong, S.P., and Harbottle, R.P. (2011). Non-viral episomal modification of cells using S/MAR elements. Expert Opin. Biol. Ther. *11*, 1177–1191.

6. Stehle, I.M., Postberg, J., Rupprecht, S., Cremer, T., Jackson, D.A., and Lipps, H.J. (2007). Establishment and mitotic stability of an extra-chromosomal mammalian replicon. BMC Cell Biol. *8*, 33.

7. Argyros, O., Wong, S.P., Fedonidis, C., Tolmachov, O., Waddington, S.N., Howe, S.J., Niceta, M., Coutelle, C., and Harbottle, R.P. (2011). Development of S/MAR minicircles for enhanced and persistent transgene expression in the mouse liver. J. Mol. Med. (Berl.) *89*, 515–529.

8. Bigger, B.W., Tolmachov, O., Collombet, J.M., Fragkos, M., Palaszewski, I., and Coutelle, C. (2001). An *araC*-controlled bacterial *cre* expression system to produce DNA minicircle vectors for nuclear and mitochondrial gene therapy. J. Biol. Chem. *276*, 23018–23027.

9. Luke, J., Carnes, A.E., Hodgson, C.P., and Williams, J.A. (2009). Improved antibiotic-free DNA vaccine vectors utilizing a novel RNA based plasmid selection system. Vaccine *27*, 6454–6459.

10. Luke, J.M., Vincent, J.M., Du, S.X., Gerdemann, U., Leen, A.M., Whalen, R.G., Hodgson, C.P., and Williams, J.A. (2011). Improved antibiotic-free plasmid vector design by incorporation of transient expression enhancers. Gene Ther. *18*, 334–343.

11. Carnes, A.E., Hodgson, C.P., Luke, J.M., Vincent, J.M., and Williams, J.A. (2009). Plasmid DNA production combining antibiotic-free selection, inducible high yield fermentation, and novel autolytic purification. Biotechnol. Bioeng. *104*, 505–515.

12. Williams, J.A., Carnes, A.E., and Hodgson, C.P. (2009). Plasmid DNA vaccine vector design: impact on efficacy, safety and upstream production. Biotechnol. Adv. *27*, 353–370.

13. Piechaczek, C., Fetzer, C., Baiker, A., Bode, J., and Lipps, H.J. (1999). A vector based on the SV40 origin of replication and chromosomal S/MARs replicates episomally in CHO cells. Nucleic Acids Res. *27*, 426–428.

14. Ansari, D., Tingstedt, B., Andersson, B., Holmquist, F., Sturesson, C., Williamsson, C., Sasor, A., Borg, D., Bauden, M., and Andersson, R. (2016). Pancreatic cancer: yesterday, today and tomorrow. Future Oncol. *12*, 1929–1946.

15. Rahib, L., Smith, B.D., Aizenberg, R., Rosenzweig, A.B., Fleshman, J.M., and Matrisian, L.M. (2014). Projecting cancer incidence and deaths to 2030: the unexpected burden of thyroid, liver, and pancreas cancers in the United States. Cancer Res. *74*, 2913–2921.

16. Malvezzi, M., Bertuccio, P., Rosso, T., Rota, M., Levi, F., La Vecchia, C., and Negri, E. (2015). European cancer mortality predictions for the year 2015: does lung cancer have the highest death rate in EU women? Ann. Oncol. *26*, 779–786.

17. Fullerton, P.T., Jr., Creighton, C.J., and Matzuk, M.M. (2015). Insights into SMAD4 loss in pancreatic cancer from inducible restoration of TGF-β signaling. Mol. Endocrinol. *29*, 1440–1453.

18. Brandl, M., Seidler, B., Haller, F., Adamski, J., Schmid, R.M., Saur, D., and Schneider, G. (2010). IKKα controls canonical TGFβ-SMAD signaling to regulate genes expressing SNAIL and SLUG during EMT in Panc1 cells. J. Cell Sci. *123*, 4231–4239.

19. Pardali, K., Kurisaki, A., Morén, A., ten Dijke, P., Kardassis, D., and Moustakas, A. (2000). Role of Smad proteins and transcription factor Sp1 in p21$^{Waf1/Cip1}$ regulation by transforming growth factor-β. J. Biol. Chem. *275*, 29244–29256.

20. Moustakas, A., and Kardassis, D. (1998). Regulation of the human p21/WAF1/Cip1 promoter in hepatic cells by functional interactions between Sp1 and Smad family members. Proc. Natl. Acad. Sci. USA *95*, 6733–6738.

21. Yasutome, M., Gunn, J., and Korc, M. (2005). Restoration of Smad4 in BxPC3 pancreatic cancer cells attenuates proliferation without altering angiogenesis. Clin. Exp. Metastasis *22*, 461–473.

22. David, C.J., Huang, Y.H., Chen, M., Su, J., Zou, Y., Bardeesy, N., Iacobuzio-Donahue, C.A., and Massagué, J. (2016). TGF-β tumor suppression through a lethal EMT. Cell *164*, 1015–1030.

23. Liu, C.Y., Lin, H.H., Tang, M.J., and Wang, Y.K. (2015). Vimentin contributes to epithelial-mesenchymal transition cancer cell mechanics by mediating cytoskeletal organization and focal adhesion maturation. Oncotarget *6*, 15966–15983.

24. Câmara, J., and Jarai, G. (2010). Epithelial-mesenchymal transition in primary human bronchial epithelial cells is Smad-dependent and enhanced by fibronectin and TNF-α. Fibrogenesis Tissue Repair *3*, 2.

25. Deer, E.L., González-Hernández, J., Coursen, J.D., Shea, J.E., Ngatia, J., Scaife, C.L., Firpo, M.A., and Mulvihill, S.J. (2010). Phenotype and genotype of pancreatic cancer cell lines. Pancreas *39*, 425–435.

26. Bode, J., Kohwi, Y., Dickinson, L., Joh, T., Klehr, D., Mielke, C., and Kohwi-Shigematsu, T. (1992). Biological significance of unwinding capability of nuclear matrix-associating DNAs. Science *255*, 195–197.

27. Stehle, I.M., Scinteie, M.F., Baiker, A., Jenke, A.C., and Lipps, H.J. (2003). Exploiting a minimal system to study the epigenetic control of DNA replication: the interplay between transcription and replication. Chromosome Res. *11*, 413–421.

28. Rupprecht, S., Hagedorn, C., Seruggia, D., Magnusson, T., Wagner, E., Ogris, M., and Lipps, H.J. (2010). Controlled removal of a nonviral episomal vector from transfected cells. Gene *466*, 36–42.

29. Hagedorn, C., Baiker, A., Postberg, J., Ehrhardt, A., and Lipps, H.J. (2012). A colony-forming assay for determining the establishment efficiency of S/MAR-containing nonviral episomal expression vectors. Cold Spring Harb. Protoc. *2012*, 706–708.

30. Noll, E.M., Eisen, C., Stenzinger, A., Espinet, E., Muckenhuber, A., Klein, C., Vogel, V., Klaus, B., Nadler, W., Rösli, C., et al. (2016). CYP3A5 mediates basal and acquired therapy resistance in different subtypes of pancreatic ductal adenocarcinoma. Nat. Med. *22*, 278–287.

31. Ryan, D.P., Hong, T.S., and Bardeesy, N. (2014). Pancreatic adenocarcinoma. N. Engl. J. Med. *371*, 2140–2141.

32. Herreros-Villanueva, M., Hijona, E., Cosme, A., and Bujanda, L. (2012). Mouse models of pancreatic cancer. World J. Gastroenterol. *18*, 1286–1294.

33. Xia, X., Wu, W., Huang, C., Cen, G., Jiang, T., Cao, J., Huang, K., and Qiu, Z. (2015). *SMAD4* and its role in pancreatic cancer. Tumour Biol. *36*, 111–119.

34. Kuhlmann, L., Nadler, W.M., Kerner, A., Hanke, S.A., Noll, E.M., Eisen, C., Espinet, E., Vogel, V., Trumpp, A., Sprick, M.R., and Roesli, C.P. (2017). Identification and validation of novel subtype-specific protein biomarkers in pancreatic ductal adenocarcinoma. Pancreas *46*, 311–322.

35. Yuan, W., and Varga, J. (2001). Transforming growth factor-β repression of matrix metalloproteinase-1 in dermal fibroblasts involves Smad3. J. Biol. Chem. *276*, 38502–38510.

36. Edgar, R., Domrachev, M., and Lash, A.E. (2002). Gene Expression Omnibus: NCBI gene expression and hybridization array data repository. Nucleic Acids Res. *30*, 207–210.

OMTM, Volume *17*

Supplemental Information

Novel Non-integrating DNA Nano-S/MAR Vectors Restore Gene Function in Isogenic Patient-Derived Pancreatic Tumor Models

Matthias Bozza, Edward W. Green, Elisa Espinet, Alice De Roia, Corinna Klein, Vanessa Vogel, Rienk Offringa, James A. Williams, Martin Sprick, and Richard P. Harbottle

Supplementary Figure 1

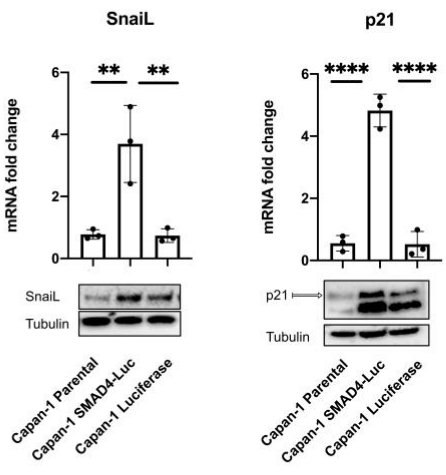

Supplementary Figure 1. Quantitative Real Time PCR analysis of SnaiL and p21 in Capan-1. The expression of SnaiL and p21 was addressed in genetically modified Capan-1 with SMAD4 and Luciferase, in comparison to parental unmodified cells through qRT-PCR and Western Blot.

Supplementary Figure 2

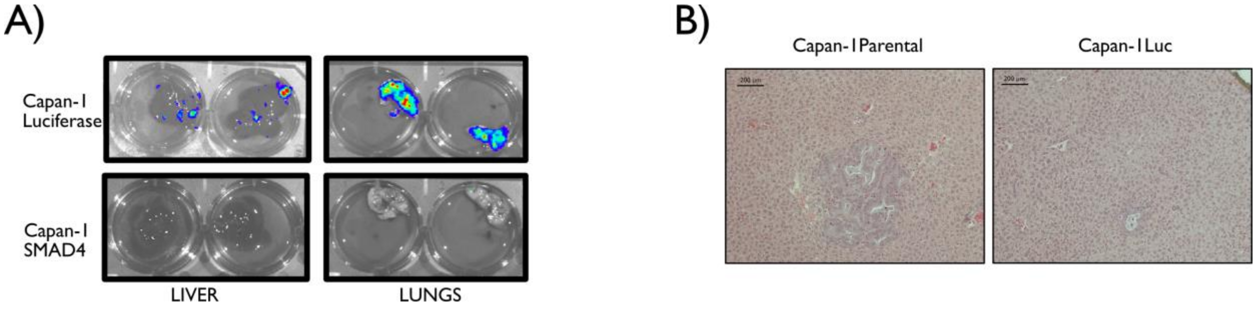

Supplementary Figure 2. Bioluminescent detection of metastasis. (A) The formation of metastasis in the liver and in the lungs of mice injected with Capan-1 labelled with the reporter gene Luciferase were detected through Bio Luminescent Imaging (BLI) imaging and (B) their morphology was assessed through H&E staining.

Supplementary Figure 3

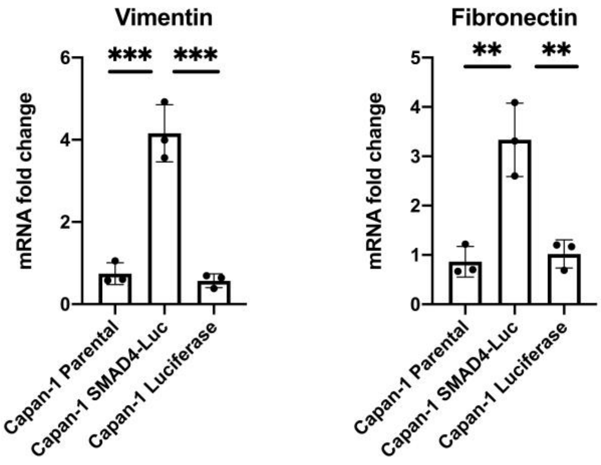

Supplementary Figure 3. Analysis of EMT markers. The expression of Vimentin and Fibronectin in modified and unmodified Capan-1 cells was investigated through qRT-PCR.

Supplementary Figure 4

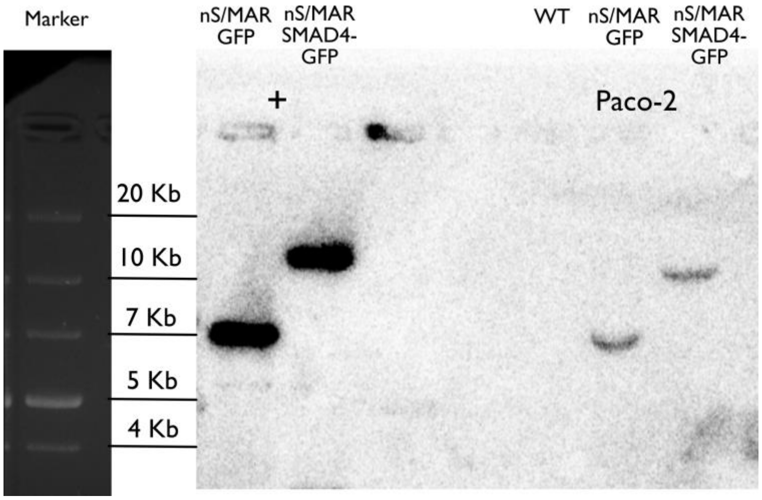

Supplementary Figure 4. Southern Blot analysis of PACO2 cells modified with Nano-S/MAR vectors.

The molecular integrity and the episomal maintenance of the two Nano-S/MAR constructs were evaluated by Southern Blot analysis. Control Nano-Vectors (first two lanes) and the total DNA

from the modified PACO2 cells was digested with the restriction enzyme BamHI and separated on a 1 % agarose gel before being transferred on a nylon membrane. The GFP reporter gene was used to generate the radioactive probe. The plasmid isolated via total DNA extraction from established cells showed an identical size to their respective reference controls. The DNA molecular weight marker is shown as a reference.

Supplementary Figure 5

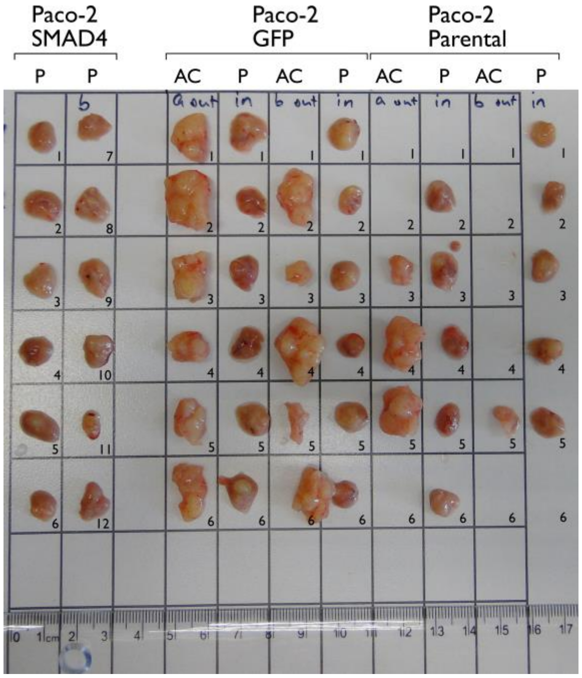

Supplementary Figure 5. PACO2 cells macroscopic evaluation of tumour growth.

PACO2 Parental and GFP cells form tumours in the pancreas (P) and in the abdominal cavity (AC) of the injected mice. Paco-2 SMAD4 cells did not form clear tumour masses in the injection site and abdominal cavity.

Supplementary Figure 6

A) SMAD4 open frame cDNA

ATGGACAATATGTCTATTACGAATACACCAACAAGTAATGATGCCTGTCTGAGCATTGTGCATAGTTTGA
TGTGCCATAGACAAGGTGGAGAGAGTGAAACATTTGCAAAAAGAGCAATTGAAAGTTTGGTAAAGAAG
CTGAAGGAGAAAAAAGATGAATTGGATTCTTTAATAACAGCTATAACTACAAATGGAGCTCATCCTAGT
AAATGTGTTACCATACAGAGAACATTGGATGGGAGGCTTCAGGTGGCTGGTCGGAAAGGATTTCCTCAT
GTGATCTATGCCCGTCTCTGGAGGTGGCCTGATCTTCACAAAAATGAACTAAAACATGTTAAATATTGTC
AGTATGCGTTTGACTTAAAATGTGATAGTGTCTGTGTGAATCCATATCACTACGAACGAGTTGTATCACC
TGGAATTGATCTCTCAGGATTAACACTGCAGAGTAATGCTCCATCAAGTATGATGGTGAAGGATGAATA
TGTGCATGACTTTGAGGGACAGCCATCGTTGTCCACTGAAGGACATTCAATTCAAACCATCCAGCATCCA
CCAAGTAATCGTGCATCGACAGAGACATACAGCACCCCAGCTCTGTTAGCCCCATCTGAGTCTAATGCTA
CCAGCACTGCCAACTTTCCCAACATTCCTGTGGCTTCCACAAGTCAGCCTGCCAGTATACTGGGGGGCAG
CCATAGTGAAGGACTGTTGCAGATAGCATCAGGGCCTCAGCCAGGACAGCAGCAGAATGGATTTACTG
GTCAGCCAGCTACTTACCATCATAACAGCACTACCACCTGGACTGGAAGTAGGACTGCACCATACACACC
TAATTTGCCTCACCACCAAAACGGCCATCTTCAGCACCACCCGCCTATGCCGCCCCATCCCGGACATTACT
GGCCTGTTCACAATGAGCTTGCATTCCAGCCTCCCATTTCCAATCATCCTGCTCCTGAGTATTGGTGTTCC
ATTGCTTACTTTGAAATGGATGTTCAGGTAGGAGAGACATTTAAGGTTCCTTCAAGCTGCCCTATTGTTA
CTGTTGATGGATACGTGGACCCTTCTGGAGGAGATCGCTTTTGTTTGGGTCAACTCTCCAATGTCCACAG
GACAGAAGCCATTGAGAGAGCAAGGTTGCACATAGGCAAAGGTGTGCAGTTGGAATGTAAAGGTGAA
GGTGATGTTTGGGTCAGGTGCCTTAGTGACCACGCGGTCTTTGTACAGAGTTACTACTTAGACAGAGAA
GCTGGGCGTGCACCTGGAGATGCTGTTCATAAGATCTACCCAAGTGCATATATAAAGGTCTTTGATTTGC
GTCAGTGTCATCGACAGATGCAGCAGCAGGCGGCTACTGCACAAGCTGCAGCAGCTGCCCAGGCAGCA
GCCGTGGCAGGAAACATCCCTGGCCCAGGATCAGTAGGTGGAATAGCTCCAGCTATCAGTCTGTCAGCT
GCTGCTGGAATTGGTGTTGATGACCTTCGTCGCTTATGCATACTCAGGATGAGTTTTGTGAAAGGCTGG
GGACCGGATTACCCAAGACAGAGCATCAAAGAAACACCTTGCTGGATTGAAATTCACTTACACCGGGCC
CTCCAGCTCCTAGACGAAGTACTTCATACCATGCCGATTGCAGACCCACAACCTTTAGAC

B) Illumina probe for SMAD4 transcript

GCAGCGTCACTCTACCTAATGTCTCACTGTTCTGCAAAGGTGGCAATGCT

Supplementary Figure 6. Sequences for microarray analysis

A) SMAD4 open reading frame was codon-optimized and stop codon was removed to allow the tandem expression of the tumour suppressor with a reporter gene. This sequence is not recognized by the Illumina probe (B) responsible for the detection of SMAD4 endogenous transcript.

TABLE

Table 1. Differentially expressed genes in Capan-1 Luciferase *vs* Capan-1 unmodified parental cells with a fold change greater than 2 (*n* = 351)

Table 2. Differentially expressed genes in Capan-1 SMAD4 Luciferase *vs* Capan-1 Luciferase cells (*n* = 189 genes)

Table 3. Differentially expressed genes in PACO2 GFP Vs PACO2 Parental cells with a fold change greater than 2 (n = 2)

Table 4. Differentially expressed genes in PACO2 GFP-SMAD4 *vs* PACO2 GFP cells with a fold change greater than 2 (n = 169)

REFERENCES

1. Dinca, EB, Sarkaria, JN, Schroeder, MA, Carlson, BL, Voicu, R, Gupta, N*, et al.* (2007). Bioluminescence monitoring of intracranial glioblastoma xenograft: response to primary and salvage temozolomide therapy. *J Neurosurg* **107**: 610-616.
2. Argyros, O, Wong, SP, Niceta, M, Waddington, SN, Howe, SJ, Coutelle, C*, et al.* (2008). Persistent episomal transgene expression in liver following delivery of a scaffold/matrix attachment region containing non-viral vector. *Gene Ther* **15**: 1593-1605.
3. Argyros, O, Wong, SP, Gowers, K, and Harbottle, RP (2012). Genetic modification of cancer cells using non-viral, episomal S/MAR vectors for in vivo tumour modelling. *PLoS One* **7**: e47920.
4. Wong, SP, Argyros, O, Coutelle, C, and Harbottle, RP (2011). Non-viral S/MAR vectors replicate episomally in vivo when provided with a selective advantage. *Gene Ther* **18**: 82-87.
5. Argyros, O, Wong, SP, and Harbottle, RP (2011). Non-viral episomal modification of cells using S/MAR elements. *Expert Opin Biol Ther* **11**: 1177-1191.
6. Stehle, IM, Postberg, J, Rupprecht, S, Cremer, T, Jackson, DA, and Lipps, HJ (2007). Establishment and mitotic stability of an extra-chromosomal mammalian replicon. *BMC Cell Biol* **8**: 33.
7. Argyros, O, Wong, SP, Fedonidis, C, Tolmachov, O, Waddington, SN, Howe, SJ*, et al.* (2011). Development of S/MAR minicircles for enhanced and persistent transgene expression in the mouse liver. *J Mol Med (Berl)* **89**: 515-529.
8. Bigger, BW, Tolmachov, O, Collombet, JM, Fragkos, M, Palaszewski, I, and Coutelle, C (2001). An araC-controlled bacterial cre expression system to produce DNA minicircle vectors for nuclear and mitochondrial gene therapy. *J Biol Chem* **276**: 23018-23027.
9. Luke, J, Carnes, AE, Hodgson, CP, and Williams, JA (2009). Improved antibiotic-free DNA vaccine vectors utilizing a novel RNA based plasmid selection system. *Vaccine* **27**: 6454-6459.
10. Luke, JM, Vincent, JM, Du, SX, Gerdemann, U, Leen, AM, Whalen, RG*, et al.* (2011). Improved antibiotic-free plasmid vector design by incorporation of transient expression enhancers. *Gene Ther* **18**: 334-343.
11. Carnes, AE, Hodgson, CP, Luke, JM, Vincent, JM, and Williams, JA (2009). Plasmid DNA production combining antibiotic-free selection, inducible high yield fermentation, and novel autolytic purification. *Biotechnol Bioeng* **104**: 505-515.
12. Williams, JA, Carnes, AE, and Hodgson, CP (2009). Plasmid DNA vaccine vector design: impact on efficacy, safety and upstream production. *Biotechnol Adv* **27**: 353-370.
13. Piechaczek, C, Fetzer, C, Baiker, A, Bode, J, and Lipps, HJ (1999). A vector based on the SV40 origin of replication and chromosomal S/MARs replicates episomally in CHO cells. *Nucleic Acids Res* **27**: 426-428.
14. Noll, EM, Eisen, C, Stenzinger, A, Espinet, E, Muckenhuber, A, Klein, C*, et al.* (2016). CYP3A5 mediates basal and acquired therapy resistance in different subtypes of pancreatic ductal adenocarcinoma. *Nat Med* **22**: 278-287.

15. Hagedorn, C, Baiker, A, Postberg, J, Ehrhardt, A, and Lipps, HJ (2012). A colony-forming assay for determining the establishment efficiency of S/MAR-containing nonviral episomal expression vectors. *Cold Spring Harb Protoc* **2012**: 706-708.
16. Ansari, D, Tingstedt, B, Andersson, B, Holmquist, F, Sturesson, C, Williamsson, C*, et al.* (2016). Pancreatic cancer: yesterday, today and tomorrow. *Future Oncol* **12**: 1929-1946.
17. Rahib, L, Smith, BD, Aizenberg, R, Rosenzweig, AB, Fleshman, JM, and Matrisian, LM (2014). Projecting cancer incidence and deaths to 2030: the unexpected burden of thyroid, liver, and pancreas cancers in the United States. *Cancer Res* **74**: 2913-2921.
18. Malvezzi, M, Bertuccio, P, Rosso, T, Rota, M, Levi, F, La Vecchia, C*, et al.* (2015). European cancer mortality predictions for the year 2015: does lung cancer have the highest death rate in EU women? *Ann Oncol* **26**: 779-786.
19. Fullerton, PT, Jr., Creighton, CJ, and Matzuk, MM (2015). Insights Into SMAD4 Loss in Pancreatic Cancer From Inducible Restoration of TGF-beta Signaling. *Mol Endocrinol* **29**: 1440-1453.
20. Brandl, M, Seidler, B, Haller, F, Adamski, J, Schmid, RM, Saur, D*, et al.* (2010). IKK(alpha) controls canonical TGF(ss)-SMAD signaling to regulate genes expressing SNAIL and SLUG during EMT in panc1 cells. *J Cell Sci* **123**: 4231-4239.
21. Pardali, K, Kurisaki, A, Moren, A, ten Dijke, P, Kardassis, D, and Moustakas, A (2000). Role of Smad proteins and transcription factor Sp1 in p21(Waf1/Cip1) regulation by transforming growth factor-beta. *J Biol Chem* **275**: 29244-29256.
22. Moustakas, A, and Kardassis, D (1998). Regulation of the human p21/WAF1/Cip1 promoter in hepatic cells by functional interactions between Sp1 and Smad family members. *Proc Natl Acad Sci U S A* **95**: 6733-6738.
23. Yasutome, M, Gunn, J, and Korc, M (2005). Restoration of Smad4 in BxPC3 pancreatic cancer cells attenuates proliferation without altering angiogenesis. *Clin Exp Metastasis* **22**: 461-473.
24. David, CJ, Huang, YH, Chen, M, Su, J, Zou, Y, Bardeesy, N*, et al.* (2016). TGF-beta Tumor Suppression through a Lethal EMT. *Cell* **164**: 1015-1030.
25. Liu, CY, Lin, HH, Tang, MJ, and Wang, YK (2015). Vimentin contributes to epithelial-mesenchymal transition cancer cell mechanics by mediating cytoskeletal organization and focal adhesion maturation. *Oncotarget* **6**: 15966-15983.
26. Camara, J, and Jarai, G (2010). Epithelial-mesenchymal transition in primary human bronchial epithelial cells is Smad-dependent and enhanced by fibronectin and TNF-alpha. *Fibrogenesis Tissue Repair* **3**: 2.
27. Deer, EL, Gonzalez-Hernandez, J, Coursen, JD, Shea, JE, Ngatia, J, Scaife, CL*, et al.* (2010). Phenotype and genotype of pancreatic cancer cell lines. *Pancreas* **39**: 425-435.
28. Bode, J, Kohwi, Y, Dickinson, L, Joh, T, Klehr, D, Mielke, C*, et al.* (1992). Biological significance of unwinding capability of nuclear matrix-associating DNAs. *Science* **255**: 195-197.
29. Stehle, IM, Scinteie, MF, Baiker, A, Jenke, AC, and Lipps, HJ (2003). Exploiting a minimal system to study the epigenetic control of DNA replication: the interplay between transcription and replication. *Chromosome Res* **11**: 413-421.
30. Rupprecht, S, Hagedorn, C, Seruggia, D, Magnusson, T, Wagner, E, Ogris, M*, et al.* (2010). Controlled removal of a nonviral episomal vector from transfected cells. *Gene* **466**: 36-42.

31. Ryan, DP, Hong, TS, and Bardeesy, N (2014). Pancreatic adenocarcinoma. *N Engl J Med* **371**: 2140-2141.
32. Herreros-Villanueva, M, Hijona, E, Cosme, A, and Bujanda, L (2012). Mouse models of pancreatic cancer. *World J Gastroenterol* **18**: 1286-1294.
33. Xia, X, Wu, W, Huang, C, Cen, G, Jiang, T, Cao, J*, et al.* (2015). SMAD4 and its role in pancreatic cancer. *Tumour Biol* **36**: 111-119.
34. Kuhlmann, L, Nadler, WM, Kerner, A, Hanke, SA, Noll, EM, Eisen, C*, et al.* (2017). Identification and Validation of Novel Subtype-Specific Protein Biomarkers in Pancreatic Ductal Adenocarcinoma. *Pancreas* **46**: 311-322.
35. Yuan, W, and Varga, J (2001). Transforming growth factor-beta repression of matrix metalloproteinase-1 in dermal fibroblasts involves Smad3. *J Biol Chem* **276**: 38502-38510.
36. Edgar, R, Domrachev, M, and Lash, AE (2002). Gene Expression Omnibus: NCBI gene expression and hybridization array data repository. *Nucleic Acids Res* **30**: 207-210.
